# Advances in Genome Editing and Application to the Generation of Genetically Modified Rat Models

**DOI:** 10.3389/fgene.2021.615491

**Published:** 2021-04-20

**Authors:** Vanessa Chenouard, Séverine Remy, Laurent Tesson, Séverine Ménoret, Laure-Hélène Ouisse, Yacine Cherifi, Ignacio Anegon

**Affiliations:** ^1^CHU Nantes, Inserm, Centre de Recherche en Transplantation et Immunologie, UMR 1064, ITUN, Université de Nantes, Nantes, France; ^2^genOway, Lyon, France; ^3^CHU Nantes, Inserm, CNRS, SFR Santé, Inserm UMS 016, CNRS UMS 3556, Nantes Université, Nantes, France

**Keywords:** CRISPR-Cas9, rat, knockout, knockin, transgenesis, genetic diseases, immune genes

## Abstract

The rat has been extensively used as a small animal model. Many genetically engineered rat models have emerged in the last two decades, and the advent of gene-specific nucleases has accelerated their generation in recent years. This review covers the techniques and advances used to generate genetically engineered rat lines and their application to the development of rat models more broadly, such as conditional knockouts and reporter gene strains. In addition, genome-editing techniques that remain to be explored in the rat are discussed. The review also focuses more particularly on two areas in which extensive work has been done: human genetic diseases and immune system analysis. Models are thoroughly described in these two areas and highlight the competitive advantages of rat models over available corresponding mouse versions. The objective of this review is to provide a comprehensive description of the advantages and potential of rat models for addressing specific scientific questions and to characterize the best genome-engineering tools for developing new projects.

## Introduction

Genetically modified animal models are essential to answering questions in biology, modeling human and non-human animal diseases, and generating therapeutic recombinant proteins. Among animal models, small laboratory mammals are often used because they share many biological features with humans, housing them is easy and relatively inexpensive compared to maintenance of large animals, and ethical issues are less prominent than with species such as non-human primates.

Among the small laboratory animal models, the rat has been used since at least 1856 (Philipeaux, 1856) and still is an important experimental model (between 9 and 18% of all laboratory models in the EU, The Commission to the European Parliament and the Council, 2015-2017).

Certain intrinsic characteristics of the rat, such as its larger size (10 fold) compared to the mouse, allow easier and more rapid microsurgery, multiple sampling of larger blood and tissue volumes, precise injection of substances into the brain, and *in vivo* and *ex vivo* organ function analysis. Additionally, mice and rats differ in their physiology and more sophisticated traits in the rat have made it a model of choice for toxicology, complex human diseases and neurobehavioral as well as cardiovascular studies among several others (Jacob, 2010).

Such differences have been supported by comparative analyses of the rat and mouse genomes. The rat genome is 2.75 gigabases (Gb), smaller than the human genome (2.9 Gb) but larger than the mouse genome (2.6 Gb) (Gibbs et al., 2004). Overall, rats show enrichment of genes involved in immunity, metabolic detoxification and chemosensation, as well as conservation of many genes involved in human diseases (Dewey et al., 2004; Gibbs et al., 2004).

**FIGURE 1:**
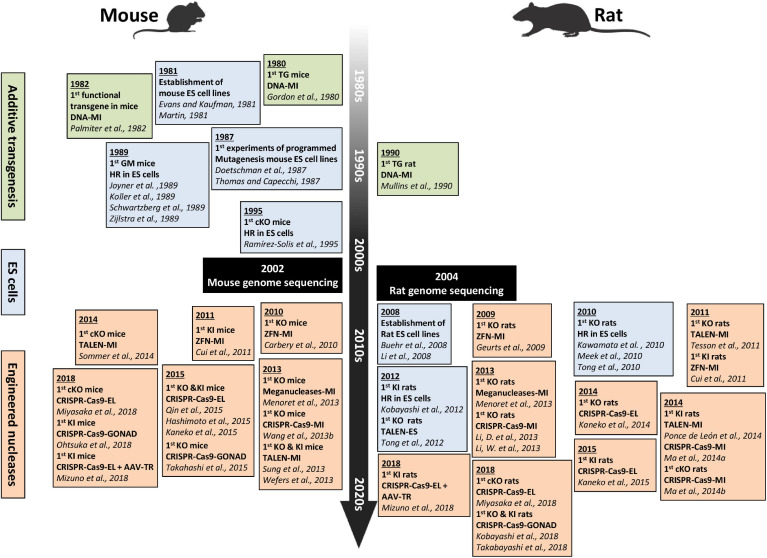
Timeline showing the major technical advances in genome editing and delivery in mice and rats from the 1980s to today. The *green frames* encompass the 1st transgenic mice and rats generated by DNA microinjection. The *blue frames* contain the 1st ES cells-based mouse and rat models, and the *orange frames* contain the 1st mouse and rat models generated using engineered nucleases delivered by different methods. Figure created with BioRender.com. AAV-TR, AAV transduction; cKO, conditional KO; DNA-MI, DNA microinjection; EL, electroporation; ES, embryonic stem cells; GM, genetically modified; GONAD, genome-editing via oviductal nucleic acids delivery; HR, homologous recombination; KI, knockin; KO, knockout; LV-MI, lentiviral microinjection; TALEN-MI, TALE nucleases microinjection; TG, transgenic; ZFN-MI, ZFN microinjection.

Despite these advantages, the use of rats has lagged behind the use of mice in research, mainly because genetically modified mice were generated earlier than genetically modified rats (Figure 1). In mice, DNA microinjection was used in the early 1980s and embryonic stem (ES) cells in the late 1980s (Gordon et al., 1980; Palmiter et al., 1982; Doetschman et al., 1987). In contrast, in rats, DNA microinjection and ES cells began in the early 1990s and 2010, respectively (Mullins et al., 1990; Kawamata and Ochiya, 2010). In the meantime, researchers used classical breeding approaches to develop a variety of rat strains that model human diseases (Szpirer, 2020). The need for genetic engineering tools for the rat and the continuous use of zygote pronuclei microinjection of DNA in the rat, explain why gene-specific nucleases were applied in rats in 2009, earlier than in mice (2010) (Geurts et al., 2009; Carbery et al., 2010). These gene-specific nucleases quickly facilitated the exponential generation of knockout (KO) rats for many genes. In synergy with these technological advances, sequencing of the rat genome (Dewey et al., 2004; Gibbs et al., 2004) and characterization of genetic quantitative trait loci (QTLs) linked to diseases (Aitman et al., 2010, 2016) further accelerated the use of models of genetically modified rats.

In this regard, different rat strains are prone to different diseases present in humans and reproduce better than mice some of these diseases. These rat strains have been used to introduce genetic modifications to analyze the role of genes (Aitman et al., 2010, 2016). For example, Wistar Kyoto, Dahl/SS, and spontaneously hypertensive strains develop hypertension and have extensively used to analyze the role of many genes (Moreno et al., 2011; Rudemiller et al., 2014; Nayak et al., 2015; Aitman et al., 2016; Lerman et al., 2019; Szpirer, 2020). The diabetes-prone biobreeding rat strain is another model that has been used to genetically modify genes involved in diabetes (Michalkiewicz et al., 2004; Pandey and Dvorakova, 2020). Lewis rats are more susceptible than mice to the induction of Th1-mediated autoimmune diseases, whereas Brown Norway rats are highly susceptible to Th2-mediated immune diseases. Genomic linkage analysis allowed identification of a region on chromosome 9 that controls these phenotypes (Bernard et al., 2010). Additionally, the rat has been extensively used to analyze autoimmune diseases involving multiple genes (Aitman et al., 2010; Bernard et al., 2010).

In this review, we first describe the evolution and advances in genome editing and in delivery optimization of CRISPRs for producing genetically modified models. Further details are given on the rat to highlight needs and future research paths. The second part of the review focuses on the advantages of genetically modified rat models compared to mouse to mimic human situation, in particular in genetic diseases and immunology studies. Rats differ from mice in several characteristics, manifesting different phenotypes for the same genetic alteration. Rats also can sometimes better reproduce clinical features observed in humans who carry these gene variants (Hammer et al., 1990; Larcher et al., 2014). Our final aim is thus to inform researchers about major progresses in rat genome editing and advantages of rats as model organisms, to give researchers the choice of the best experimental system to answer their scientific questions. To facilitate rat models access and development, major rat resources for finding existing models or designing new ones with the latest gene editing tools, are described in Table 1.

**TABLE 1:** Resources on rat genomics and genome edited animals.

**Resources**	**Name**	**Website and references**	**Proposed resources**
**Genomic databases**	National Center for Biotechnology Information (NCBI) including Gene, Protein, Nucleotide, Blast, and others	www.ncbi.nlm.nih.gov/ (Sayers et al., 2019)	Comprehensive suite for molecular analysis from rat genome to protein expression and functionality
	The European Bioinformatics Institute (EMBL-EBI) including Ensembl, UniProt, Clustal Omega and others	https://www.ebi.ac.uk/services (Madeira et al., 2019)	From rat genome to protein databases a full suite with analysis tools and multiple sequence alignments
	The University of California, Santa Cruz Genome Browser	https://genome.ucsc.edu/ (Lee et al., 2020)	Genome browser, multiple sequence alignments and others
	Model organism Aggregated Resources for Rare Variant exploration (MARRVEL)	http://marrvel.org/ (Wang et al., 2019b)	Comparison of human genes with model oragnisms’ genes such as the rat in a physiologic or pathologic context
**Genomic databases and strains repository**	Rat Genome Database (RGD) in the United States	https://rgd.mcw.edu (Smith et al., 2020)	Repository of hundreds or rat strains and genome edited rats, mostly for genes involved in hypertension and cardiovascular function. Genetic, phenotype and disease data, sequences, QTLs, mapping data, software tools.
**Rat strains repository**	Rat Resource and Research Center (RRRC) in the United States	http://www.rrrc.us/	Repository of hundreds or rat strains, genome edited lines, cryopreserved embryos, sperm, and ES cells.
	National Bioresource Project for the rat (NBPR) in Japan	http://www.anim.med.kyoto-u.ac.jp/nbr/	Repository of hundreds or rat strains, ENU and genome edited lines, cryopreserved embryos and sperm, BAC libraries
	Rat Resource Database in China	http://www.ratresource.com	Repository of rat strains and genomic data.
	Rodent Model Research in Taiwan	https://www.nlac.narl.org.tw/	Strain depository of lines or rats including genome edited ones.
**Academic platforms producing genome-edited rat models**	Wisconsin Gene Editing Rat Resource Center and The Michigan University Transgenic Animal Core facility in the United States	https://rgd.mcw.edu/wg/gerrc/ https://brcf.medicine.umich.edu/cores/transgenic-animal-model/	Distribution of already available models and generation of new ones on demand
	Transgenic Rat ImmunoPhenomic (TRIP) facility in France	http://www.itun.nantes.inserm.fr/Core-facilities/TRIP-Transgenic-Rats-ImmunoPhenomic	
**Commercial vendors for rat models**	Charles River laboratories	https://www.criver.com/	Distribution of already available models and generation of new ones on demand
	Janvier Labs	https://www.janvier-labs.com/	
	Envigo (include Horizon discovery models)	https://www.envigo.com/research-models	
	Taconic Biosciences	https://www.taconic.com	
	genOway (include Axenis models)	https://www.genoway.com/	
	Cyagen	https://www.cyagen.com/us/en/	Custom rat model generation
	Hera Biolabs	https://www.herabiolabs.com/ SRG OncoRats (Noto et al., 2020)	Proprietary gene editing technologies and SRG OncoRats for oncology studies
	Ligand pharmaceuticals	https://www.ligand.com/technologies/omniab OmniRat (Joyce et al., 2020) OmniFlic (Harris et al., 2018)	OmniRat and OmniFlic for human antibodies generation
**Software for the use of CRISPR**	CRISPOR	http://crispor.tefor.net/ (Concordet and Haeussler, 2018)	On and off target scores
	CHOPCHOP	https://chopchop.cbu.uib.no/ (Labun et al., 2019)	
	E-CRISPR	http://www.e-crisp.org/E-CRISP/ (Heigwer et al., 2014)	
	CCTOP	https://cctop.cos.uni-heidelberg.de:8043/index.html (Stemmer et al., 2015; Labuhn et al., 2018)	
	CRISPRscan	https://www.crisprscan.org/ (Moreno-Mateos et al., 2015)	
	CRISPRdirect	http://crispr.dbcls.jp/ (Naito et al., 2015)	Off-target prediction only
	CRISPR RGEN tools	http://www.rgenome.net/	Cas-OFFinder, Microhomology, Cas-designer, base-editing, prime-editing…
**Private company webtool for design of gRNA targeting rat genome**	Integrated DNA Technologies	https://eu.idtdna.com/pages/products/crispr-genome-editing	Include on and off target scores
	Synthego	https://www.synthego.com/products/bioinformatics/crispr-design-tool	
	Horizon Discovery	https://horizondiscovery.com/en/ordering-and-calculation-tools/crispr-design-tool	
	Benchling	https://www.benchling.com/crispr/	

## Gene-Editing Advances and Delivery System Optimization

**FIGURE 2:**
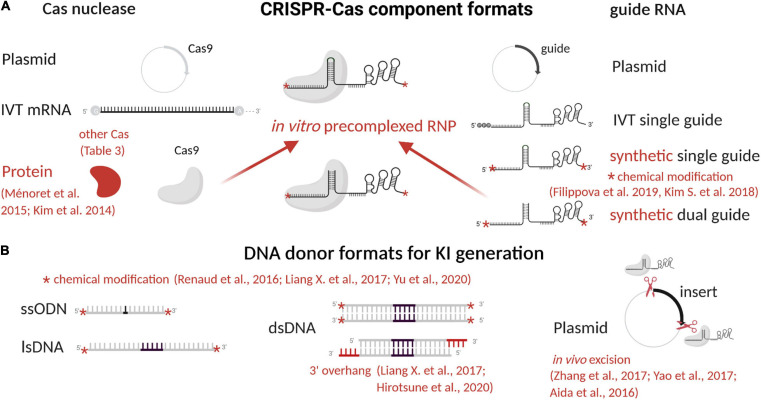
CRISPR-Cas9 component formats and advances to enhance editing efficiency. **(A)** CRISPR-Cas9 consists of a Cas9 nuclease and a gRNA that can be used in different formats (plasmid, mRNA, or protein) to form the RNP complex. **(B)** A DNA donor can also be used to generate KI models, also in different formats (ssODN, lsDNA, plasmid, dsDNA). In *red* are indicated advances to enhance efficacies of editing. Other Cas used for rodent models generation are described in **Table 3**. Figure created with BioRender.com. IVT, *in vitro* transcribed; RNP, ribonucleoprotein complex; DSB, double-strand break; ssODN, single-stranded oligonucleotide; lsDNA, long single-stranded DNA; dsDNA, linear double-stranded DNA.

**FIGURE 3:**
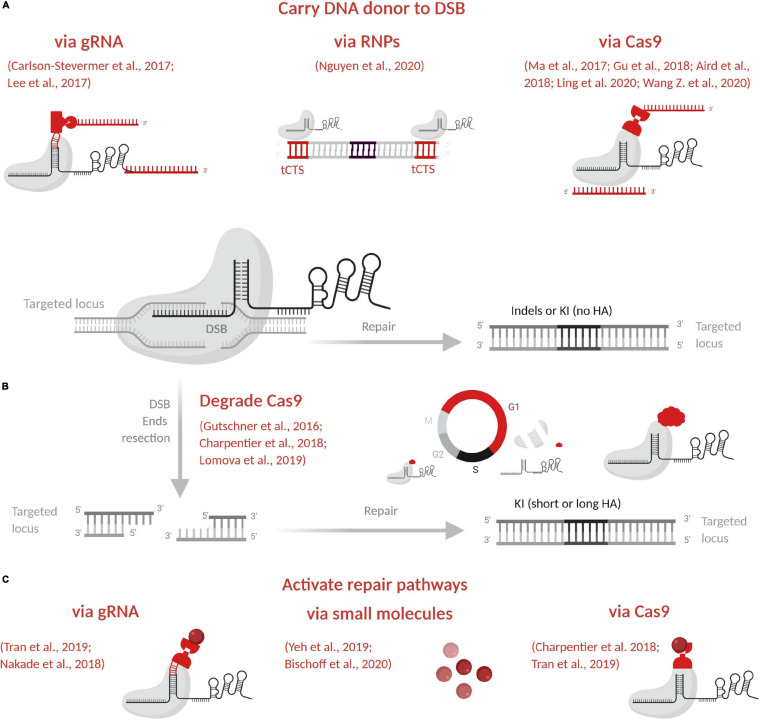
Promising strategies to enhance KI model generation. **(A)** Carry DNA donor to the DSB via gRNA, via the RNP complex or via Cas9. **(B)** Degrade Cas9 by the proteasome in G1 to favor homology-directed repair pathways predominant in S/G2. **(C)** Activate homology-directed repair pathways via gRNA, via small molecules or via Cas9. In *red* are indicated and illustrated the main approaches to enhance editing efficacy. Figure created with BioRender.com. DSB, double-strand break; indels, insertions or deletions; KI, knockin; HA, homology arms; gRNA, guide RNA; RNP, ribonucleoprotein complex; tCTS, truncated Cas9 target sequences.

The last four decades have brought major advances in genome editing allowing for generation of animal models that harbor targeted genetic modifications. Efforts have focused on increasing the precision of these modifications, production efficiency and on simplifying procedures to make them easier and cheaper. The evolution of genome editing approaches and tools is discussed in this section, illustrated in Figure 1 and nucleases compared in Table 2. Clustered, regularly interspaced short palindromic repeat (CRISPR)-associated (Cas) systems applied to rodents are detailed in Table 3, with details of specifics regarding rats given in this section. More particularly, *Streptococcus pyogenes* (SpCas) system components are described in Figure 2 and compared in Table 4. Published advances for enhancing knockin (KI) generation rate are also detailed here and illustrated in Figure 3. Finally, delivery systems and the evolution of their practice are detailed and compared in Table 5.

**TABLE 2:** Comparison of engineered endonucleases.

**Specificities, advantages, limitations**	**Meganucleases**	**ZFN**	**TALEN**	**CRISPR-Cas**
DNA binding determinant	Protein	ZF protein	TAL protein	crRNA/sgRNA

Binding specificity	Long sequences of nucleotides^*a*^	3 nucleotides	1 nucleotide^*b*^	1/1 nucleotide pairing

Endonuclease	I-CreI and I-SceI^*a*^	FokI^*c*^	FokI^*c*^	Cas9

Function specificity	Monomer	Dimer	Dimer	Monomer

Design/Engineering	Very difficult	Difficult	Simple	Very simple

Restriction in target site	Chromatin compaction	G-rich sequence	Start with T and end with A	End with a NGG sequence

Target site length	18–44 bp	18–36 bp^*d*^	24–40 bp	22–25 bp

Targeting frequency	Low	High (one/100 bp)	High (one/bp)	High (one/4 or 8 bp)

Specificity	High	Moderate^*e*^	High	High

Sensitivity to DNA methylation	Yes	Yes	Yes	No^*f*^

Off-targets	Variable	Low^*e*^	Very low	Variable

Size	Small size	Small size (∼1 kb/monomer)	Large size (∼3 kb/monomer)	Large size (4.2 kb Cas9)

Commercially available, Cost	Yes, high	Yes, high	Yes, moderate	Yes, low

Patents concern	Yes	Yes	Yes	Yes

**Type of editing**				

Gene KO (Indels and frameshift)	Yes	Yes	Yes	Yes

Multiplex KO	No data^*h*^	Very limited	Limited	Yes (up to eight alleles)^*g*^

Gene correction/point mutagenesis (repaired basepairs)	No data^*h*^	Yes	Yes	Yes

Gene addition/sequence replacement (integrated gene cassette)	No data^*h*^	Yes	Yes	Yes

Gene deletion (deleted gene fragments)	No data^*h*^	No data	No data	Yes

Prime and base editing	No data^*h*^	No data	No data	Yes

*^*a*^DNA-binding specificities and cleavage mechanism combined in the same protein (Galetto et al., 2009). I-CreI and I-SceI are the main endonucleases used but a few others have been applied to genome editing. ^*b*^TALE protein consist of 34 amino acid repeat domains, each one recognizing a single DNA nucleotide; highly conserved, excepting two hypervariable residues at positions 12 and 13, which confer the specificity of TALE. ^*c*^FokI cleaves only in its dimeric form ^*d*^Association of 3–6 ZF DNA binding domains fused to the FokI catalytic domain. Binding of two ZFN-FokI heterodimers to two contiguous DNA sequences and separated by a 5–7 bp gap. ^*e*^Specificity depends on number and selected ZF modules. ^*f*^No direct effect of methylation on Cas9 binding or effectivity (Verkuijl and Rots, 2019). ^*g*^Difficult on same chromosome. Limitations overcome by Prime and base editing (cf Table 3). ^*h*^The difficulty in designing meganucleases has limited their application in creating new model organisms.*

**TABLE 3:** CRISPR variants applied to genetically modified mouse and rat models.

**Application**	**Type – Variant - Name**	**PAM 5′-3′**	**Cleavage**	**GM mice**	**GM rats**
**Classical GE**	II- SpCas9	NGG	Blunt DSB	Wang et al., 2013b	Li D. et al., 2013; Li W. et al., 2013
**Specificity enhancement**	II- E -Hypa SpCas9	NGG	Blunt DSB	Ikeda et al., 2019	^–^
	II- E -SpCas9 nickase	NGG	Nick	Ran et al., 2013	^–^
**Enlarge targeting possibilities**	II- E -SpCas9 VQR	NGA	Blunt DSB	Robertson et al., 2018	^–^
	II- E -SpCas9 VRER	NGCG	Blunt DSB	Robertson et al., 2018	^–^
	II- E -SpCas9-NG	NGN	Blunt DSB	Fujii et al., 2019	^–^
	II- SaCas9	NNGRRT	Blunt DSB	Zhang X. et al., 2016	Zheng et al., 2020
	II- E -SaCas9 KKH	NNNRRT	Blunt DSB	Robertson et al., 2018	^–^
	II- St1Cas9	NNAGAAW	Blunt DSB	Fujii et al., 2016	^–^
	II- CjCas9	NNNVRYM	Blunt DSB	Kim et al., 2017	^–^
	II- NmCas9	NNNNGATT	Blunt DSB	Xia et al., 2018	^–^
	II- FnCas9	NGG	5′ staggered	Hirano et al., 2016	^–^
	V-A- AsCpf1 (Cas12a)	TTTV	5′ staggered	Hur et al., 2016; Kim et al., 2016	Lee J. G. et al., 2019; Yeo et al., 2019
	V-A- LbCpf1 (Cas12a)	TTTV	5′ staggered	Kim et al., 2016	Lee J. G. et al., 2019
	V-A- ErCas12a CRISPR-Mad7	TTTN, CTTN	5′ staggered	Liu Z. et al., 2020	Liu Z. et al., 2020
	V-A- CRISPR-Mb3Cas12a	TTV	5′ staggered	Wang Z. et al., 2020	^–^
	V-B- AaCas12b (C2c1)	TTN	5′ staggered	Teng et al., 2018	^–^
**Alternative editing**	**Cytosine base editing** II- E -SpBE2 II- E -HF2-SpBE2 II- E -SpBE3 II- E -Sp-BE4 II- E -Sp-VQR-BE3 II- E -SaBE3	NGG from NGG/A to NGG NGG NGG NGA NNGRRT	None None Nick Nick Nick Nick	Lee et al., 2018 Liang P. et al., 2017 Zhang H. et al., 2018 Lee et al., 2018 Lee et al., 2018 Liu et al., 2018	- - - - - -
	**Adenosine base editing** II- E -SpABE7.10 II- E -SpVQR-ABE II- E -SaKKH-ABE	NGG NGA NNNRRT	Nick Nick Nick	Liu et al., 2018 Yang L. et al., 2018 Yang L. et al., 2018	Yang L. et al., 2018 ^−^ ^−^
	**Prime editing** PE3	NGG	2 Nicks	Liu Y. et al., 2020	^–^

*GE, genome editing; E, engineered Cas; GM, genetically modified model; DSB, double strand break; St1Cas9, *Streptococcus thermophilus* Cas9; CjCas9, *Campylobacter jejuni* Cas9; NmCas9, *Neisseria meningitidis* Cas9; FnCas9, *Francisella novicida* Cas9.*

**TABLE 4:** CRISPR-Cas9 component format advantages, limits and advances.

**Format**	**Advantages**	**Limitations**	**Advances demonstrated in any species (rat in bold)**
**Cas9**			

**Plasmid**	No limit on insert size Easy engineering High expression	Delayed activity Mosaicism Increased off-targets Delayed activity	**Cas9 protein allowing rapid and more efficient editing** (Kim et al., 2014; Ménoret et al., 2015) Large editing toolbox variants **(Table 3)** Improved chromatin accessibility (Chen F. et al., 2017; Ding et al., 2019) **Cas9 engineered to activate repair pathways** (Charpentier et al., 2018; Tran et al., 2019) Cas9 engineering to be degraded in G1 (Gutschner et al., 2016; Charpentier et al., 2018; Lomova et al., 2019)
**mRNA**	Expression faster than plasmid Limit mosaicism and off-targets	Delayed activity *In vitro* transcription efficiency/toxicity	
**Protein**	Ready to cut Limit mosaicism and off-targets Affordable and high quality	Crystallization at high dose *In vivo* stability potentially immunogenic	

**gRNA**			

**Plasmid**	No limit on insert size Easy to engineer	Delayed activity	Chemical modification (Kim S. et al., 2018; Filippova et al., 2019) Essential sequence, secondary structures and functional modules of gRNA (Briner et al., 2014; Kartje et al., 2018) Overlapping gRNA (Jang et al., 2018) gRNA engineering to activate repair pathways (Nakade et al., 2018; Tran et al., 2019)
**IVT sgRNA**	Easy to produce and use Flexible in sequence and length Efficient	Time-consuming production Induced immune responses Limited in chemical modification	
**Synthetic sgRNA**	Affordable and high quality Chemical modifications Ready to use Efficient	Order full sgRNA for each project Long RNA synthesis Difficulties in adding fluorophore for tracking	
**Synthetic dgRNA**	Short RNA synthesis Low cost and high quality Same tracrRNA for all project Chemical modifications Fluorophores added for tracking Efficient	crRNA & tracrRNA hybridization *in vitro*	

**DNA donor**			

**ssODN**	Low cost synthesis High efficacy for mutation or short KI	Limited in length to 200nt	DNA synthesis progresses (Hao et al., 2020) **Chemical modification** (Renaud et al., 2016; Liang X. et al., 2017; Yu et al., 2020) Insertion close to cut site (Inui et al., 2014; Liang X. et al., 2017) 3′ overhang DNA donor (Liang X. et al., 2017; Hirotsune et al., 2020) Carry to cut site by Cas9 (Ma et al., 2017; Aird et al., 2018; Gu et al., 2018; Ling et al., 2020; Wang Z. et al., 2020) Carry to cut site by gRNA (Carlson-Stevermer et al., 2017; Lee et al., 2017) Carry to cut site by DNA donor engineering (Nguyen et al., 2020) DNA donor in vivo excision from plasmid (Aida et al., 2016; Yao et al., 2017; Zhang et al., 2017)
**lsDNA**	Usable for long KI	Limited in length Difficult to produce Mutated KI Expensive to synthesize	
**dsDNA**	Usable for long KI Easy to produce and engineer No limit on insert size	Few random insertions	
**Plasmid**	Usable for long KI Easy to produce and engineer No limit on insert size	Few random insertions	

*IVT, *in vitro*–transcribed; gRNA, guide RNA; sgRNA, single gRNA; dgRNA, dual gRNA; ssODN, single-stranded oligonucleotides; lsDNA, long single-stranded DNA; dsDNA, linear double-stranded DNA.*

**TABLE 5:** Delivery methods.

**Delivery methods**	**Cargo**	**Species/cell target**	**Location**	**Advantages**	**Limitations**	**References**
**Physical delivery**						

**Microinjection**	DNA donor - dsDNA (linear/plasmid) - dsDNA encoding gene-specific nucleases - lsDNA (>200nt) - ssODN (∼100nt)	Mouse and rat zygote	Pronucleus or cytoplasm	- Delivery of large DNA fragments - Stable DNA in cell	- Time-consuming method - Expertise required (less for Cyt-MI) - Poor visualization pronucleus, flexibility of the oolemma and nuclear membranes in rat - Variability in efficiency depending on size, DNA quality or purity - Persistent expression and depending on host transcriptional/transductional machinery	1st description (Gordon et al., 1980; Palmiter et al., 1982; Mullins et al., 1990) dsDNA-ZFN (Geurts et al., 2009) dsDNA-TALEN (Tesson et al., 2011) dsDNA-Meganuclease (Ménoret et al., 2013) Efficiency (Charreau et al., 1996b; Hirabayashi et al., 2001) Complex/invasive method (Brinster et al., 1985; Charreau et al., 1996b)
	mRNA encoding gene specific nucleases	Mouse and rat zygote	Pronucleus or cytoplasm	- Moderate efficiency - Transient expression - Cyt-MI more efficient than PN-MI - Off-target reduced - Independent expression dependency of host transcriptional/transductional machinery (mRNA)	- Time-consuming - Expertise required (less for Cyt-MI) - Variation among batches of IVT mRNA - mRNA liable to degradation	mRNA-ZFN (Geurts et al., 2009) mRNA-TALEN (Tesson et al., 2011; Remy et al., 2014) mRNA-CRISPR (Ménoret et al., 2015) Meganucleases (Wang et al., 2014)
	Protein (RNP)	Mouse and rat zygote Mouse/ES	Pronucleus or cytoplasm	- Higher efficiency than using DNA or mRNA encoding gene specific nucleases - Short half-life within cells - Less mosaicism - Off-target cleavage reduced	-*In vivo* stability -Potentially immunogenic	(Ménoret et al., 2015; Wang et al., 2015; Jung C. J. et al., 2017)

**Electroporation**	DNA donor - dsDNA (linear/plasmid) - ssODN - lssDNA (600–1.5 kb)	Mouse and rat zygote	Uncontrolled cytoplasm (long DNA) Pronucleus (short lsDNA/ssODN)	- Easier delivery than DNA-MI - Processing simultaneously 50–60 zygotes in a short time - Efficient to deliver ssODN or lsDNA (<1 kb)	- Inefficient nuclear transport - Transient nuclear envelop breaking or cell-division required - Inefficient to deliver DNA > 1 kb	ssODN (Hashimoto and Takemoto, 2015; Kaneko and Mashimo, 2015; Qin et al., 2015; Chen et al., 2016; Wang et al., 2016; Remy et al., 2017) lsDNA (Miyasaka et al., 2018) Inefficient delivery dsDNA (Takabayashi et al., 2018)
	mRNA encoding Cas9 + sgRNA	Mouse and rat zygote	Uncontrolled	- Easier delivery than mRNA-MI	- Embryos are quite sensitive to pulse and toxicity is observed	Rat/mRNA encoding Cas9+sgRNA (Remy et al., 2017) CRISPR/mice/KO/HDR-KI (Qin et al., 2015) Mice/CRISPR/KO (Hashimoto and Takemoto, 2015; Hashimoto et al., 2016) Rat/ZFN/TALEN/Crispr/KO (Kaneko et al., 2014; Kaneko and Nakagawa, 2020) Rat/mice/Crispr/KO/KI (Kaneko and Nakagawa, 2020)
	Protein (RNP)	Mouse and rat zygote	Uncontrolled	- Easier delivery than RNP-MI	- High amount of cargo - Uncontrolled delivery amount	Cas9-RNP/mice/indels/large KO/HDR-KI/ssODN-KI (Wang et al., 2016) Cas9-RNP/mice/KO (Hashimoto et al., 2016)
**GONAD**	DNA - ssODN - lsDNA (<1 kb)/ Cas9 mRNA/sgRNA RNP	Mouse and rat	Oviduct	- *Ex vivo* embryo handling steps not required - Fewer animals used (e.g., recycling females possible)	- Not yet applicable to deliver long donor DNA (db or long ss DNA)	Cas9mRNA + sgRNA/mice/KO (Takahashi et al., 2015) RNP/lsDNA/mice/KO/ssODN and lsDNA-based KI (Ohtsuka et al., 2018) Rat/ssODN based KI (Kobayashi et al., 2018; Takabayashi et al., 2018)

**Viral delivery methods**						

**AAV vectors (Non-enveloped, lsDNA)**	DNA encoding Cas9/sgRNA (separate AAV or all-in-one AAV) – KI DNA cassette	Mouse and rat zygote (transduction)	Uncontrolled	- minimal immunogenicity - low toxicity - wide-range serotypes - No incorporation into the host genome	Low capacity (<5 Kb)	KO/Mice/separate AAV (Yoon et al., 2018) KO/KI/Mice/Rat/RNP Electroporation/AAVtransduction (Mizuno et al., 2018; Chen et al., 2019) (Edraki et al., 2019)
	DNA (expression cassette)	Mouse zygote microinjection	Cytoplasmic injection			(Yu et al., 2015)

*MI, microinjection; Cyt-MI, cytoplasmic microinjection; PN-MI, pronuclear microinjection; DNA-MI, DNA microinjection; KI, Knockin; ssODN, single-stranded oligonucleotides; lsDNA, long single-stranded DNA; dsDNA, linear double-stranded DNA; HDR-KI, homology directed repair knockin; RNP, ribonucleoprotein complex.*

### Historical Overview of Major Gene-Editing Techniques Developed in Mice and Rats

#### Random Additive Transgenesis and Mutagenesis

The first transgenic rodents were successfully generated in the early 1980s and 1990s (Gordon et al., 1980; Palmiter et al., 1982; Mullins et al., 1990), by microinjection of exogenous donor DNA into the pronucleus of one-cell embryos. The reported efficiencies are quite low in rodents, ranging from 0.5 to 10% of injected embryos in mice and 0.5–5% of injected embryos in rats (Brinster et al., 1985; Charreau et al., 1996b; Hirabayashi et al., 2001). Other problems include random integration, a high copy number of integrated DNA sequences in *cis* and uncontrollable transgene expression. These challenges make this approach labor intensive and time-consuming and require considerable expertise.

*N*-ethyl-*N*-nitrosurea (ENU) is a highly potent mutagen that was first administered into adult male mice (Bode, 1984) and later into rats (Zan et al., 2003). Several ENU-induced mutant rat (van Boxtel et al., 2010) (for a review see Huang et al., 2011) and mouse models (for a review see Justice et al., 1999) have been described. This method presents some advantages: it requires no embryos or ES handling and the sperm of mutant offspring can be cryopreserved. Disadvantages include uncontrolled and random mutations in multiple loci throughout the genome, which must be identified and localized using high-throughput and time-consuming screening methods.

Transposon-mediated insertional transgenesis is an alternative tool developed to increase the integration frequency of the transgene into the host genome. Transposons are simple and mobile elements, consisting of a DNA sequence encoding transposase and a transgene flanked by binding sites (inverted terminal repeats, ITR) for the transposase, promoting integration into the genome. Transposon systems, such as *Sleeping Beauty* (SB), piggyBac (PB) or Tol2, have demonstrated their efficiency in rapidly producing stable lines of transgenic mice (Carlson et al., 2003; Horie et al., 2003) and rats (Kitada et al., 2007; Lu et al., 2007). The number of transgene insertions is, however, difficult to control.

#### Targeted Mutagenesis

The derivation of germline-competent mouse ES cells in the early 1980s (Evans and Kaufman, 1981; Martin, 1981) and the first experiments of targeted mutagenesis (Doetschman et al., 1987; Thomas and Capecchi, 1987), allowed introducing mutations into the host genome with a high precision (Joyner et al., 1989; Koller et al., 1989; Schwartzberg et al., 1989; Zijlstra et al., 1989) making mice a privileged model for genetic studies for two decades. Rat ES cells were described in 2008 (Buehr et al., 2008; Li et al., 2008) allowing generation of KO (Kawamata and Ochiya, 2010; Meek et al., 2010; Tong et al., 2010) and KI rats (Kobayashi et al., 2012; Yamamoto et al., 2015) with similar homologous recombination (HR) efficiencies to those observed in mice. Nevertheless, rat ES cells are less robust than mouse ES cells and maintaining their stability in culture and germline competence continues to be challenging.

The development of meganucleases, engineered zinc-finger nucleases (ZFNs), transcription activator-like effector nucleases (TALENs) and more recently the CRISPR-Cas system, has unquestionably revolutionized genome editing, opening new possibilities especially in the rat and other species in which ES cells were not available (Fernández et al., 2017). Each of these nucleases have their own properties of DNA-binding, recognition type/site specificities, their own advantages and limitations, which are listed in Table 2. Injection of these nucleases directly into rat or mouse zygotes allows creation of a double-strand break (DSB) at a targeted locus, repaired thereafter mainly by non-homologous end-joining (NHEJ) or HR (these mechanisms are reviewed in detail in a later section). Careful design of the associated tools makes it possible to better control repair outcome at any targeted locus of the genome with high efficiency and much faster than with ES cells. Several reports demonstrated the high efficiency of ZFN and TALEN in quickly generating different types of modifications in mice and rats, ranging from KO (Geurts et al., 2009; Carbery et al., 2010; Mashimo et al., 2010, 2013; Tesson et al., 2011; Tong et al., 2012; Sung et al., 2013; Sommer et al., 2014), simple point mutations, to large KI by homology-directed repair (HDR) (Sung et al., 2013; Wang et al., 2013a; Wefers et al., 2013; Ponce de León et al., 2014; Remy et al., 2014). Meganucleases, although less used than the other nucleases, were also applied to generate KO mouse and rats (Ménoret et al., 2013). Nevertheless, the design complexity and associated costs made these techniques accessible to only few laboratories, leading to a search for alternative approaches.

The simplicity and rapidity of guided RNA design, compared to complex protein engineering needed for ZFNs and TALENs, made the CRISPR-Cas system largely accessible at low cost, without sacrificing the specificity and reproducibility already observed with ZFNs and TALENs. Nevertheless, the success of CRISPR-Cas, especially in the generation of the first CRISPR mouse (Wang et al., 2013b) and rat (Li D. et al., 2013; Li W. et al., 2013), depended on knowledge gathered using the previous gene-specific nucleases in terms of DNA cleavage outcomes, repair pathways mechanisms (molecules involved and forms of DNA donors) and genotyping techniques.

#### CRISPR-Cas Systems

The CRISPR-Cas9 system is originally based on a ribonucleoprotein (RNP) complex composed of a nuclease (Cas9) driven by a dual-guide RNA (dgRNA) duplex (Jiang and Doudna, 2017). Cas9 cleavage capacity relies on its two nuclease domains, each cleaving one strand of the genomic DNA. Inactivation of either nuclease domain (nickase) generates a nick on the corresponding strand (Jinek et al., 2012), whereas inactivation of both domains (dead Cas9 or dCas9) completely abolishes its cleavage capacity. The native dgRNA (Deltcheva et al., 2011) is formed from a trans-activating CRISPR RNA (tracrRNA) harboring a complex secondary structure to interact with Cas9 and a CRISPR RNA (crRNA), that mostly encodes the 20 nucleotides that give the system its specificity. When formed, this RNP complex quickly interrogates genomic DNA for its specific protospacer adjacent motif (PAM). The PAM is a key factor because it defines the possibilities of DNA targeting sequences. For SpCas9, the targets are limited to a G-rich genomic region with a 5′-NGG-3′ PAM (Jinek et al., 2014; Nishimasu et al., 2014). PAM recognition is followed by specific gRNA (guide RNA) spacer (20 nucleotides) matching. A perfect match creates a targeted blunt DSB three nucleotides away from the PAM. A few mismatches between the gRNA and the targeted genomic DNA are tolerated at certain positions and may lead to off-target editing (Peng et al., 2018). Design of gRNA with the highest homology specificity possible for the targeted DNA sequence is essential to limit off-target edits (Ayabe et al., 2019). Available tools for rat genome editing with CRISPRs are described in Table 1. Off-target is less of an issue for animal model generation when compared to the use of gene editing as a therapeutic tool. Indeed, animals require multiple breeding, clearing lines from off-targets on chromosomes different from the one harboring the mutation of interest.

To expand the CRISPR toolbox, many variants of SpCas9 have been engineered and bacterial strains screened to either enhance specificity or broaden PAM opportunities. Variants (Pickar-Oliver and Gersbach, 2019) and SpCas9 ortholog classification (Makarova et al., 2020) have been recently reviewed. Many of these options have been used at least once to edit mouse embryos, but only a few have been applied to the rat. Those already applied to rodent genome editing are summarized in Table 3. Type V Cas have T-rich PAMs and other interesting features, such as staggered DSB generation, that make them complementary to SpCas9. For this reason, some orthologs of Cpf1 (Cas12a) are the most used after SpCas9, including *Acidaminococcus* sp. (AsCpf1) (Lee J. G. et al., 2019; Yeo et al., 2019) and *Lachnospiraceae bacterium ND2006* (LbCpf1) (Lee J. G. et al., 2019).

Classical genome editing, alternatives and their context of application have been recently reviewed in detail (Anzalone et al., 2020). Two of these, namely base editing and prime editing, have been used for rodent genome editing and are summarized in Table 3. Cytosine base editor has been engineered using either dCas9 or nickase to transform cytosine into a thymine (Komor et al., 2016; Nishida et al., 2016) and was further improved (Rees and Liu, 2018; Schatoff et al., 2019). Adenine base editor was engineered to mutate adenine into guanine more efficiently than Cas9 genome editing in human cells (Gaudelli et al., 2017). Several base editor variants have been applied to mouse embryos for single (Liang P. et al., 2017) or multiple (Liu et al., 2018; Zhang H. et al., 2018) base editing, whereas only the SpABE7.10 system has been applied in rats (Ma Y. et al., 2018; Yang L. et al., 2018). The main advantage of base editing is its capacity to generate targeted indels or a particular mutation without a DNA donor, enhancing its efficiency compared to classical genome editing. By avoiding DSBs, this system also allows multiplex editing on the same region of a chromosome (Lee H.K. et al., 2019). Its major limitations are bystander effect on non-targeted bases, cytosine and adenine limitations, targeted precision that restrict possibilities, and off-target effects as with classical genome editing. Prime editing is overcoming some of these limitations (Anzalone et al., 2019). This system allows mutation, short insertion and short deletion editing with limited indels generation in contrast to classical Cas genome editing. The first two versions of this system relied on a Cas9 nickase fused to a reverse transcriptase and a prime editing gRNA (pegRNA). This system induces nicking on the non-target strand and reverse transcription of the template encoded in the pegRNA to specifically modify the targeted locus. Prime editing 3 and 3b have been enhanced by the use of a second nickase with its own guide RNA, to target the strand that was not nicked by the pegRNA. Very recently, prime editing 3 has been successfully applied to genetically modify mouse embryos for the first time (Liu Y. et al., 2020). This particularly interesting approach will be applied eventually to generate genetically modified rat models.

### Advances in CRISPR-Cas Production and Design for Rodent Genome Editing

The components of the CRISPR-Cas system, both for KO or KI, have been closely studied and enhanced to increase efficiency, decrease side effects, and offer better control over repair outcomes, as reviewed below. In particular, we summarized CRISPR-Cas9 component formats and their evolution in Table 4 and Figure 2, and advances to increase KI efficiency are illustrated in Figure 3.

#### RNP Complex

KO and KI model’s generation mainly depends on RNP complex cleavage efficiency. Many studies have been done to find RNP complex best settings. It has been clearly demonstrated that the use of Cas9 protein allows transient and faster editing (Kim et al., 2014) necessary for proper animal model generation and increases efficiency of the RNP complex in mouse and rat zygotes (Figure 2A and Table 4) (Ménoret et al., 2015). Guide RNA’s sequence has been extensively studied to better understand its flexibility and structure (Table 4) (Briner et al., 2014; Kartje et al., 2018) for improved efficacy. In cells, the 5′ triphosphate group on *in vitro*–transcribed gRNA induces the cell immune system and reduces editing efficacy. This reaction can be limited by phosphatase treatment or prevented by chemical modification of synthetic gRNA (Kim S. et al., 2018). Chemical modifications and gRNA optimization have been recently reviewed (Filippova et al., 2019) and offer a clear advantage for synthetic gRNA (Figure 2A and Table 4). Regarding their format, both dgRNA and single gRNA (sgRNA) display similar efficiency (Terao et al., 2016; Shapiro et al., 2020). Chromatin state can influence editing efficiency (Janssen et al., 2019; Verkuijl and Rots, 2019) and even prevent editing of gRNA with predicted high on target score. Two main strategies have been developed in cells only to open chromatin locally and increase editing efficiency with SpCas9 and other orthologs (Table 4). The first approach uses one or multiple dCas molecules to open chromatin in close proximity to the targeted locus (Chen F. et al., 2017). The second approach relies on fused chromatin-modulating peptides on SpCas9 and other Cas proteins (*Streptococcus pasteurianus* Cas9, *Campylobacter jejuni* Cas9, and others) (Ding et al., 2019). This field is still emerging and requires further studies. There is a need for better understanding of genome editing hurdles to allow edits at any locus with high efficiency.

#### DNA Donor

DNA donors have been used in different formats to generate KI models: plasmids, single-stranded oligonucleotides (ssODNs), long single-stranded (ls)DNA, and linear double-stranded (ds)DNA (Figure 2B and Table 4). These formats and their design are important to direct repair toward KI. Because efficient KI generation is the most important issue currently, here we review the main aspects and advances regarding the DNA repair template and pathways.

Historically, transgenesis (Gordon and Ruddle, 1982; Palmiter et al., 1982; Mullins et al., 1990; Charreau et al., 1996b) and targeted mutagenesis using nucleases have been achieved using circular plasmids or an excised dsDNA, to introduce a complete expression cassette in rat and mouse genome (Cui et al., 2011; Brown et al., 2013). DNA synthesis advances in recent decades (Hao et al., 2020) have supported progress in genome editing (Table 4), allowing efficient synthesis of dsDNA, ssODNs and lsDNA, with increasing size and purity from commercial vendors. Nevertheless, yield issues persist with synthesis of long DNA fragments. Today, short sequence insertion and precise mutations are mostly generated using ssODNs. Its current synthesis limit is 200 nucleotides or fewer for most providers. A few years ago, lsDNA emerged as a new and efficient way to generate complex KI mouse (Miura et al., 2015; Miyasaka et al., 2018) and rat (Yoshimi et al., 2016; Miyasaka et al., 2018) models. Different production strategies have been developed, including *in vitro* transcription and reverse transcription (Miura et al., 2015), plasmid excision by nicking endonucleases (Yoshimi et al., 2016) and synthesis. High yield and purity are difficult to achieve for lsDNA production, leading to unexpected mutations in addition to the desired KI genotypes (Codner et al., 2018). Synthesis is quite expensive and limited to some kilobases depending on vendors (Figure 2B and Table 4). Chemically modified ssODNs, in cells and rodents, generally lead to higher editing efficiency (Renaud et al., 2016; Liang X. et al., 2017). A study on human cells showed increased KI efficacy using 5’-end–modified dsDNA (Yu et al., 2020). The proof of concept of this protection has clearly been demonstrated and will probably be tested for all DNA donor formats.

Several approaches have been developed to optimize DNA donor design, but no clear consensus has emerged regarding impact on KI efficiency. In human cells, some donors have shown better KI efficiency with ssODN complementary to the non-target strand (Richardson et al., 2016), but others have shown similar efficacy for both designs (Liang X. et al., 2017). In the same way, studies on human cells suggest better efficiency with asymmetric ssODNs (Richardson et al., 2016), whereas others report similar KI efficiency with both asymmetric and symmetric donors in mouse embryos (Lanza et al., 2018). Furthermore, in human cells (Liang X. et al., 2017) and mouse embryos (Hirotsune et al., 2020), dsDNA with 3’ overhangs displays better KI efficiency (Figure 2B and Table 4). This improvement could be explained by necessary genomic DNA end resection for KI generation during repair pathways, as discussed later. The only consensus regarding DNA donor design is that the inserted sequence should be as close as possible to the Cas9 cut site (Table 4) to yield efficient KI (Inui et al., 2014; Liang X. et al., 2017). To avoid multiple cleavages on the KI inserted sequences, silent mutations are introduced in the DNA donor close to the PAM.

Major hurdles remain for large (long donor) or complex KI (several ssODNs with complex sequence). One clear way to increase KI efficiency is to use the RNP complex to carry the DNA donor to the DSB (Figure 3A and Table 4). In this way, all KI components will be present at the same time and concentrate at the cut site. The stable and high affinity between biotin and streptavidin (Le et al., 2019) and the easy production of biotinylated DNA donor have inspired several approaches. Cas fused with avidin and a biotinylated DNA donor has been tested to generate modified mice (Ma et al., 2017; Gu et al., 2018; Wang Z. et al., 2020). The sgRNA has also been engineered to insert a specific S1M aptamer of streptavidin and improve KI generation in human cells (Carlson-Stevermer et al., 2017). To ensure tight linkage, guide RNA and the ssODN donor have also been chemically linked to crRNA (Lee et al., 2017). Covalent attachment of the DNA donor to a Cas9 fused to porcine circovirus 2 Rep protein has been also described (Aird et al., 2018). Recently, Cas9-ssODN conjugates generated chemically or via an adaptor complementary to part of the ssODN, have been used to enhance HDR-mediated genome editing in mouse zygotes (Ling et al., 2020). Another team has used the RNP complex itself in human cells, without modifying it, but by inserting 16-nucleotide truncated Cas9 target sequences (tCTSs) in the linear dsDNA donor (Nguyen et al., 2020). This tCTSs allows RNP recognition without cleavage or use of a dCas9.

#### Repair Pathways

NHEJ is the most used pathway for DSB repair which produces indels alleles by ligase IV direct ends ligation through well-described mechanisms (Frit et al., 2019). When a DNA repair template is available at the DSB, other pathways may be induced, based on homology recognition. In contrast to NHEJ, other repair pathways, i.e., HR, microhomology-mediated end joining (MMEJ), and single-strand annealing (SSA), depend on a DNA template and are predominant in S/G2 phases. To favor KI, different strategies with small molecules have been used to arrest cells at different phase of the cycle (Yeh et al., 2019; Bischoff et al., 2020) but these strategies are difficult to apply to embryos. To favor HDR pathways predominant in S/G2, Cas9 can be degraded by the proteasome in G1 phase (Figure 3B and Table 4) by fusion to geminin degron (Gutschner et al., 2016; Charpentier et al., 2018; Lomova et al., 2019). Mouse two-cell embryos have a long G2 phase (Palmer and Kaldis, 2016) and open chromatin state that is favorable for KI model generation. Gu et al. (2018) have taken advantage of these features to develop the two-cell homologous recombination (2C-HR)-CRISPR in mouse, to increase large KI efficiency with WT Cas9 or Cas9 fused to monomeric streptavidin coupled with a biotinylated donor. This approach has been reproduced in mouse using Mb3Cas12a (Wang Z. et al., 2020).

All of these repair mechanisms except NHEJ have a key first step in common: DSB end resection (for a review, see Ranjha et al., 2018). The MRE11-RAD50-NBS1 complex must first be recruited to DSB ends, where it drives CtIP and other resection molecules (Ranjha et al., 2018). Exo1 can further resect DSB ends to produce 3′ overhangs that will be coated by replication protein A (RPA). For HR, RPA will later be replaced by Rad51 to promote strand exchange, whereas for SSA, RPA-coated resected ends are recognized by Rad52 for processing by end annealing. Factors unique for MMEJ are still unclear, but it requires short resection, necessitating the inhibition by RPA end coating. The size of this resection is linked to the repair pathway that is active. Short resection will leave a short sequence for homology-driven repair, as with MMEJ (5–25 bp) and SSA (>20 bp), whereas long resection will allow for long homology recognition, as with HR (>500 bp), and no resection will trigger NHEJ. These features drive the design of DNA donor homology arms (Yao et al., 2017).

To favor KI, small inhibitors of NHEJ or essential molecules carried to the DSB via gRNA, via Cas9 (Figure 3C and Table 4) have been used. NHEJ inhibitors have mainly been tested on cells (for reviews, see Yeh et al., 2019; Bischoff et al., 2020) and SCR7, an inhibitor of ligase IV, has led to KI increase in mouse (Maruyama et al., 2015; Singh et al., 2015) and rat embryos (Ma et al., 2016). Cas9 in fusion with a domain of CtIP has shown increased KI efficiency in human cells and rats (Charpentier et al., 2018; Tran et al., 2019). In the same way, the use of a MS2 aptamer on the gRNA to carry CtIP showed better KI efficiency in cells than other molecules (Nakade et al., 2018; Tran et al., 2019). Small molecules treatments to increase KI efficiency have been reviewed (Yeh et al., 2019; Bischoff et al., 2020). No data was reported to date in rats or mice, and only two studies showed that RS-1 enhances KI efficiency in rabbit (Song et al., 2016) and bovine embryos (Lamas-Toranzo et al., 2020). Finally, tests on cells and mouse embryos have shown that ExoI overexpression enhances KI activity (Aida et al., 2016).

CRISPR-Cas9 has a repair profile closer to the environmental DSB’s one compared to other nucleases with a high frequency of insertions of one nucleotide (Trimidal et al., 2019) and mainly repairs using out-of-frame indels (>70%) and microhomologies (Guo et al., 2018; Taheri-Ghahfarokhi et al., 2018).

One study on mouse embryos showed that multiple overlapping (at least > 5 bases) sgRNAs with ssODNs increase KI efficiency, probably by inducing shorter deletions (Jang et al., 2018) (Table 4). Several studies have designed plasmid donors with inserts flanked by gRNA recognition sites to excise it within a cell or zygote (Figure 2B and Table 4). This strategy may coordinate DSB and DNA donor availability at the cut site but can also create the same ends on both the DNA donor and the genomic DNA. It has led to increased KI in cells with various lengths of the homologous arms (Zhang et al., 2017), in mouse and monkeys embryos with HMEJ arms of 800 bp (Yao et al., 2017) or in cells and mouse embryos MMEJ homology arms of 40 bp (Aida et al., 2016). The results of these studies suggest that repair outcomes can be influenced or used to favor KI. Further experiments should be done in the rat to confirm these results.

### Delivery Strategy Overview and System Optimization

Gene-editing efficiency by targeted-mutagenesis approaches, unquestionably depends on the delivery system used. In the following section, we describe the commonly used methods and recently developed strategies, which are summarized in Table 5. Latest methods are reported in Figure 1.

#### Microinjection

Since its development in mice in the early 1980s (Gordon et al., 1980; Palmiter et al., 1982), microinjection has become the most commonly used method to introduce different cargos into mouse and rat zygotes. Pronuclear injection, is a well-established method and allows the delivery of purified nucleic acid in any form (plasmid or dsDNA, lsDNA or ssODN, mRNA, gRNA, RNP) and any size (for review, see Giraldo and Montoliu, 2001). Nevertheless, the efficiency of the method is variable, depending in particular on the quality and size of DNA sources, and also the skill of the manipulator (Charreau et al., 1996b; Hirabayashi et al., 2001). In some cases, the pronucleus is hard to visualize and the flexibility of the oolemma and nuclear membranes, as in the rat, make delivery of DNA constructs more complex and invasive (Brinster et al., 1985; Charreau et al., 1996b). Cytoplasmic injection (CI) is an alternative to overcome these technical problems and has been described to deliver linearized DNA (Brinster et al., 1985), mRNA-encoding nucleases or sgRNA (Geurts et al., 2009; Tesson et al., 2011; Remy et al., 2014; Wang et al., 2014; Ménoret et al., 2015; Doe et al., 2018), allowing for a transient expression of nucleases and thus reducing off-target events. TALEN and CRISPR-Cas in the form of proteins can also be directly injected into the zygote pronucleus, cytoplasm, or both sequentially to achieve gene modifications (KO and/or KI). For proteins, efficiencies are higher for CRISPR and lower for TALEN than those observed with delivery in their DNA or mRNA forms (Table 5; Ménoret et al., 2015; Wang et al., 2015; Jung C. J. et al., 2017).

#### Electroporation

Delivery of ZFN, TALEN, or CRISPR-Cas9 nucleic acids or protein components using zygote electroporation enables generation of mice (Hashimoto and Takemoto, 2015; Qin et al., 2015; Hashimoto et al., 2016; Wang et al., 2016) or rats (Kaneko et al., 2014; Kaneko and Mashimo, 2015; Remy et al., 2017) carrying various genetic modifications (Table 5). These modifications include NHEJ-mediated indels (Kaneko et al., 2014; Hashimoto and Takemoto, 2015; Kaneko and Mashimo, 2015; Qin et al., 2015; Hashimoto et al., 2016; Wang et al., 2016; Remy et al., 2017), large segment deletions (Hashimoto et al., 2016; Wang et al., 2016), conditional KO (Miyasaka et al., 2018), double-KO (Teixeira et al., 2018), HDR-mediated precise nucleotide substitutions (Kaneko and Mashimo, 2015; Qin et al., 2015; Wang et al., 2016) or short sequence insertions using ssODNs (typically < 200 bp) (Hashimoto and Takemoto, 2015; Chen et al., 2016; Wang et al., 2016; Remy et al., 2017) and lsDNA (from 600 bp to 1.5 kb) (Miyasaka et al., 2018). In some studies, electroporation was done in mouse zygotes that were denuded of the zona pellucida (ZP) by a Tyrod’s acid treatment (Qin et al., 2015; Chen et al., 2016; Wang et al., 2016), without affecting the early development unlike data reported in rats (Okuyama and Funahashi, 2012). Electroporation also can be applied to mouse and rat frozen zygotes for efficient introduction of CRISPR RNP complexes, without affecting embryo viability or development (Nakagawa et al., 2018; Kaneko and Nakagawa, 2020).

Electroporation is thus an excellent alternative to microinjection for genome editing in mice and rats, with similar or sometimes higher success rates. It also allows the simultaneous processing of many zygotes in a short time (e.g., a batch of 50 zygotes in few seconds) without requiring expensive equipment and operators with extensive training and expertise. Nevertheless, a major limitation is the low efficiency or even absence of efficacy of this method for introducing a large DNA fragment (>500 bp) using dsDNA; even if entry into the zygote cytoplasm is achieved, the migration into the nucleus is blocked (Remy et al., 2017). LsDNA (up to 1.5 kb) has been described as an alternative (Miyasaka et al., 2018) but with lower KI yields than those observed using short ssODNs. These results have not always been reproducible, probably because of an inefficient migration into the zygote pronucleus (Remy et al., 2017).

#### Genome Editing via Oviductal Nucleic Acid Delivery (GONAD)

GONAD has the advantages of electroporation without requiring sacrifice of embryo donor animals or *ex vivo* embryo manipulation. In this technique, the RNP complex is directly injected into the oviduct of a pregnant mouse or rat, followed by *in situ* electroporation. It was first described to generate NHEJ using Cas9 mRNA (Takahashi et al., 2015; Gurumurthy et al., 2016, 2019b) and then the improved GONAD (iGONAD) was reported by Ohtsuka et al. (2018) in mice to efficiently generate indels mutations, large deletions, and ssODN and lsDNA-based KI (up to 1 kb), by replacing Cas9 mRNA by Cas9 RNP. Other groups have demonstrated the efficiency of iGONAD in rats for gene disruption and ssODN-based KI (Kobayashi et al., 2018; Takabayashi et al., 2018) and in mice by substituting Cas9 with AsCpf1 (Ohtsuka et al., 2018) (for review see Sato et al., 2020).

#### Viral Vectors

Since efficacy of KI using long DNA donors is still low, AAV vectors have been used to deliver DNA cargo. Although AAV has a reduced packaging capacity (∼5.2 Kb), that limits their use in delivering large functional components of TALEN and SpCas9, some studies have reported AAV-mediated delivery (mainly with the serotype 6) (Ellis et al., 2013) to generate mutations in mouse and rat zygotes, by using either a dual-AAV system carrying SpCas9 and sgRNA in separate vectors (Yoon et al., 2018) or sgRNA and a shorter Cas9 ortholog in an “all-in-one” vector (Edraki et al., 2019). Two groups have also managed to generate KI mice (Mizuno et al., 2018; Chen et al., 2019) and rats (Mizuno et al., 2018) by combining zygote electroporation to deliver the RNP complex and AAV transduction to introduce a large donor dsDNA (up to 4.9 kb) with efficiency ranging from 6 to 100% depending on the viral concentration (Mizuno et al., 2018). The method has not been rigorously compared with other methods and requires generation of high-purity AAV vectors.

Sleeping Beauty and PiggyBac transposons systems have been optimized to deliver CRISPR-Cas system into cells to increase gene editing efficiency and allow multi-allele targeting (Weber et al., 2015; Xu et al., 2017; Hu et al., 2018; Ye et al., 2019). Note, however, that CRISPR-Cas integration by transposon into the genome and its long-term expression in the cells could lead to off-target effects.

#### Rat Research Models and Applications

**FIGURE 4:**
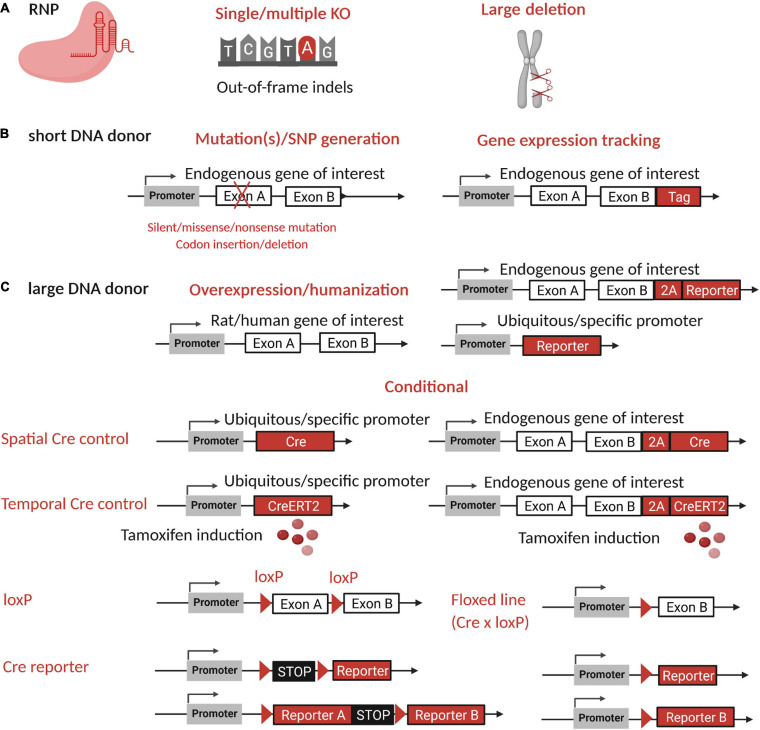
Rat research model generation by CRISPR-Cas9 and applications. Strategies to generate research models by CRISPR-cas9 are multiple and very helpful for studies of gene function and diseases or to generate a reporter model. **(A)** The RNP alone can be used to create indels at one or more loci to generate single or multiple KO or a large deletion. **(B)** RNP with a short DNA donor (ssODN) can be used to generate a stop codon or mutations or to insert a Tag in the reading frame of the endogenous gene of interest. **(C)** A large DNA donor (either lsDNA, dsDNA, or plasmid) can be used to express a reporter gene in the reading frame of the endogenous targeted gene with a self-cleaving peptide, to generate conditional or inducible Cre/lox models with or without a reporter, or to overexpress the rat or human gene of interest or a reporter gene in a safe harbor locus. For expression of inserted genes, an endogenous or ubiquitous promoter or a specific promoter can be used to restrict expression to tissues or cell types. Figure created with BioRender.com. SNP, single nucleotide polymorphism; RNP, ribonucleoprotein complex; 2A, self-cleaving peptide; KO, knockout; indels, insertion or deletion; Cre, Cre recombinase.

Today, it is possible to generate a broad range of genetically modified models, from simple KOs with precise mutations or gene overexpression, to conditional or reporter models. Below, we describe the main strategies to develop these models, which also are illustrated in Figure 4. Main resources available to find and develop rat models are available in Table 1. Table 6 describes models already developed to study genes of the immune system. Genome editing application in genetic disease studies is also explained and illustrated by the existing models listed in Table 7. Advantages of the rat as a model for those two applications are highlighted in this section.

**TABLE 6:** Genetically engineered rat models for genes of the immune system.

**(A)**					

**Immunology domain**	**Gene/genetic modification**	**Genomic tool used**	**References**	**Phenotype and rats vs. mice**	**Depository or breeder company ID**
Immuno-deficient models	*Rag1/KO* or *Rag2/*KO	Meganuclease CRISPR	Zschemisch et al., 2012; Ménoret et al., 2013; Tsuchida et al., 2014; Chang et al., 2015; Noto et al., 2018	T-B-NK+. *Rag1/*KO or Rag2/KO rats and mice show similar phenotypes	*Rag2* KO; NBRP Rat #0894
	*Foxn1/KO*	CRISPR	Goto et al., 2016	T-B+NK+. *Foxn1/*KO rats and mice show similar immune and albino phenotypes	RGD #10053598 #10053601
	*Il2rg/*KO	TALENs CRISPR	Mashimo et al., 2010; Samata et al., 2015; Kuijk et al., 2016	T-B+/-NK-. *Il2rg/*KO rats and mice show similar phenotype	#0585
	*Rag1/*KO *or Rag2/*KO *or Prkdc/*KO *or and Il2rg/*KO	ZFNs TALEN CRISPR	Mashimo et al., 2012; Ménoret et al., 2018; He et al., 2019	T-B-NK-. KO rats and mice show similar phenotypes	IL2Rg-Rag2 KO; NBRP Rat #0895 RRG (TRIP)
	*Human SIRPa/*Tg	BAC microinjection	Goto et al., 2016; Jung et al., 2016; Yang X. et al., 2018; Ménoret et al., 2020	↓ phagocytosis human cells. hSIRPa/Tg rats and mice show similar phenotype	
	*Rag1/KO or Rag2/*KO *or Prkdc/*KO *or +Il2rg/*KO*+human SIRPa/*Tg	ZFNs, TALENs, CRISPR	Yang X. et al., 2018; Ménoret et al., 2020	T-B- NK-, ↓ phagocytosis human cells Similar phenotypes in KO and Tg rats and corresponding mice as well in KO NOD mice which have a spontaneous mutation in Sirpa	RRGS (TRIP)
	*Ighm, Iglc, Igkc /*KO	ZFNs	Ménoret et al., 2010; Panzer et al., 2018	T+B-NK+. *Ighm*/KO and *IgKc*/KO rats and mice show similar phenotype	IgM KO (Ligand)
	*Human Ig heavy and/or light chain loci/Tg*	BAC microinjection	Osborn et al., 2013; Ouisse et al., 2017; Xu et al., 2018	Production of human IgG binding domains for the generation of fully human mAbs *Human Ig heavy and/or light chain loci/*Tg rats and mice show similar phenotype	Ligand
	*C3/KO*	CRISPR	Xu et al., 2018	Role of complement in neuropathy during chemotherapy model not available in mice because of defects in complement activation in mice	RGD #19165133
CDs and membrane molecules	*HLA-B27 + hb2m/*Tg	DNA microinjection	Hammer et al., 1990	*HLA-B27 + hb2m*/Tg rats are a much better model of spondyloarthropathy than are *HLA-B27 + hb2m*/Tg mice	*HLA-B27 RGD* #*7387221*
	*hCD55 + hCD59/*Tg	DNA microinjection	Charreau et al., 1996a, 1999	*hCD55 + hCD59/Tg* rat hearts were heterotopically grafted in primates Not possible for corresponding mice	/
	*hCD46/*Tg	DNA microinjection	Niewiesk et al., 1997	Model of measles infection and complement control*. hCD46*/Tg rats and mice show similar phenotypes	/
	*hCD4/hCCR5*/Tg	DNA microinjection	Keppler et al., 2002	*hCD4/hCCR5*/Tg rats are a closer model to human *hCD4/hCCR5*/Tg mice exhibited very little or no productive infection	/
	*hFasL/*Tg	DNA microinjection	Tesson et al., 1999; Bouchet et al., 2002	Expression in endothelial cells Model not available in mice	/
	*hCD21/*Tg	DNA microinjection	Yang et al., 2003	Model of EBV infection *hCD21/*Tg rats and mice show similar phenotypes	/
	*hCD64/*Tg	DNA microinjection	van Vuuren et al., 2006	Depletion of macrophages a CD64-immunotoxin and inhibition of arthritis Transgenic rats and mice have similar expression	/
	*hP2Y2R/*Tg	Lentiviral vector	Agca et al., 2009	Tissue inflammation, increase in certain leukocyte populations No hP2Y2R transgenic mouse line generated	/
	*Cd247* (CD3 ζ chain)/KO*	ZFNs	Rudemiller et al., 2014	Fewer kidney lesions in a model of hypertension similar immune phenotype in *Cd247*/KO rats and mice in T cell signaling and depletion of T cells No model of hypertension analysis in *Cd247*/KO mice	*RGD #6484582 #6484564 #6484568*
	*Tlr4*/KO	TALENs	Ferguson et al., 2013	*Tlr4*/KO rats and mice show similar decreased pro-inflammatory cytokine secretion upon lipopolysaccharide stimulation	*RRRC* #694
	*Cd40/*KO*	CRISPR	Haller et al., 2017	*Cd40/*KO rats have fewer kidney lesions in a model of hypertension than mice No model of hypertension analysis in *Cd40*/KO mice	*RRRC #840*
	*Adora2b/*KO*	ZFNs	Nayak et al., 2015	Adora2b/KO rats but not mice showed decreased pro-inflammatory cytokine secretion and less cardiac and renal injury/fibrosis in response to hypertension	RGD #6484715
	*Clec1/*KO	ZFNs	Lopez Robles et al., 2017	*Clec1*/KO rats but not mice showed increased inflammatory responses by DCs	*(TRIP)*
	*Cd59/*KO	CRISPR	Yao and Verkman, 2017b	Cd59/KO rats and not mice (showed mild hemolytic anemia and a faithful model of neuromyelitis optica	RGD #13792606
	*Kv1.3/*KO	ZFNs	Chiang et al., 2017	Kv1.3 KO rats are a better and closer model to human. Mouse T cells, unlike rat or human T cells, co-express additional redundant Kv1 channels	/
Cytokines/secreted products and their receptors	*Avp/*Tg	DNA microinjection	Jessop et al., 1995	A model for the study of thymic arginine vasopressin in T cell differentiation No analysis of AVP expression in thymus of transgenic mice	/
	*Ifng/*Tg	DNA microinjection	Egwuagu et al., 1999a,b	IFNgamma expression in the eye in a model of uveitis Conflicting results: IFN-g exacerbates uveitis in the rat and confers protection in the mouse	/
	*TGFb1/*KO*	ZFNs	Chen et al., 2013	Rats and mice TGFb1/KO with a T cell-specific deletion of the Tgfb1 gene developed lethal immunopathology in multiple organs	RGD #5131989
	*Il22bp/*KO	CRISPR	Martin et al., 2016	IL22BP protective in models of colitis and psoriasis	(TRIP)
	*Ifnar1/*KO	CRISPR	Qaisar et al., 2017	Absence of IFN-I responses *Ifnar1*/KO rats and mice not analyzed in the same way	RGD #12910493 #12910494
	*Il15/KO*	ZFNs	Renaud et al., 2017	A genetic model of NK-cell deficiency in rats *Il15/KO* rats and mice show similar phenotypes	RRRC #769
	*Tbet/KO*	ZFNs	Ma Z. G. et al., 2018	T-bet can direct Th1 lineage commitment *Tbet*/KO rats and mice show similar phenotypes	/
	*Csf1r/*KO	ES cells	Pridans et al., 2018	Absence of most macrophages in most tissues. Macrophages effects in development of multiple organ systems in rats were distinct from those reported in mice	/
	*Csf1r-GFP/*KI	DNA microinjection	Irvine et al., 2020	*Csf1r-GFP/KI* rats and mice show similar phenotypes	/
Intracellular molecules	*HMOX1/*Tg	DNA microinjection	Braudeau et al., 2003	*HMOX1/*Tg only described in rats	/
	*Hmox1/KO*	ZFNs	Atsaves et al., 2017	*Hmox1*/KO rats and mice show similar phenotype with generalized inflammation and kidney lesions and lethality	
	*Ian5/Tg*	PAC microinjection	Michalkiewicz et al., 2004	A model that shows the essential role of IAN5 for lymphoid development. IAN5 rescues lymphopenia in BB rats with a mutation in the *Ian5* gene	/
	*Notch1/Tg*	DNA microinjection	van den Brandt et al., 2005	Blockade of thymic development and T cell lymphopenia *Notch1*/Tg rats and mice show similar phenotypes	/
	Selenoprotein M/Tg	DNA microinjection	Hwang et al., 2008	Maintenance of a high level of antioxidant status Selenoprotein M/Tg rats and mice show similar phenotypes in brain	/
	*Bcl2/*Tg	DNA microinjection	Iscache et al., 2011	Increased B cells and immunoglobulins *Bcl2*/Tg rats and mice show similar phenotypes	/
	*Cyp2j4/KO*	ZFNs	Behmoaras et al., 2015	Cyp2j4 determines a profibrotic macrophage transcriptome Implications in various inflammatory conditions Similar results in *Cyp2j4/KO* rats and mice	RGD #12904679
	*Ahr/KO*	ZFNs TALENs CRISPR	Harrill et al., 2013; Phadnis-Moghe et al., 2016	A variety of T and B cell alterations. *Ahr*/KO rats are more analyzed than Ahr/KO mice Rats showed other organ alterations	RGD #12903250 (Horizon Discovery); RGD #12903272 (Horizon discovery) RGD #13838845 (not available)
					RRRC#831 (CRISPR) RGD #15090819 #15090817 (TALEN, not available)
	*Aire/*KO	ZFNs	Ossart et al., 2018	Autoimmunity in several organs *Aire*/KO rats not observed in *Aire*/KO mice	(TRIP)
	*Prox1 promoter-EGFP/Tg*	BAC microinjection	Jung E. et al., 2017	Visualization of all lymphatic vessels *Prox1 promoter-EGFP/Tg* rats and mice show similar phenotypes	/
	*Eogt/KO*	TALENs	Hao et al., 2018	*O*-GlcNAc glycosylation deficiency with defect in Notch signaling in autoimmune hepatitis *Eogt/KO* rats and mice show similar phenotypes	/
	*Paraoxonase 1/KO*	CRISPR	Bai et al., 2018	Thymocyte blockade at the CD4/CD8 double-negative to double-positive transition stage No mouse model reported	RGD #12790692 #12790698 #12790695
	*S100A8 transgenic rats/Tg*	DNA microinjection	Okada et al., 2018	Altered macrophage function in a colitis model *S100A8/Tg* rats and mice show similar phenotypes	/

**(B)**		

	**Gene/KO**				

Miscellaneous	*Snx25*/KO, *Axl/*KO*, *Cd14/*KO*, *Cd55/*KO, *Cd226/*KO, *Cyba*/KO*, *Cybb/*KO*, *Fyn/*KO*, *Gpr183*/KO*, *Ifnar1/KO*				Unpublished, available at MCW RGD		

*Performed in the Dahl/S strain. WCM RGD, Wisconsin Medical College Rat Genomic Database. EBV; Epstein Barr virus.

**TABLE 7:** Genetically modified rat models of human genetic diseases.

**System/organ affected**	**Human genetic disease**	**Gene/genetic modification**	**Genomic tool used**	**References**	**Rats vs. mice**	**Depository or breeder company ID**
**Cardiovascular**	pulmonary arterial hypertension	*BMPR2/KO*	ZFN	Ranchoux et al., 2015; Hautefort et al., 2019; Manaud et al., 2020	*Bmpr2*KO rats showed pulmonary vascular cell phenotypes closer to human patients than in*Bmpr2* KOmice	*RGD#38501086 (not available) RGD #14975305 #14981588*
	Primary pulmonary hypertension 4 (PPH4)	*Kcnk3/KO*	CRISPR-Cas9	Lambert et al., 2019	Rats have a *Kcnk3* gene as humans do but mice do not	/
	Atrial fibrillation, familial, 18 (ATFB18)	*Myl4/KO*	CRISPR-Cas9	Peng et al., 2017	This model reproduces the human disease No*Myl4/KO* mouse model is reported	/
	Familial hypertrophic cardiomyopathy and myocardial genetic diseases	Myh7b/KO	CRISPR-Cas9	Chen et al., 2020	This model reproduces the human disease No *Myh7b/KO* mouse model is reported	/
	Danon disease	*Lamp2/KO*	TALEN	Wang et al., 2017; Ma S. et al., 2018	*Lamp2*-KO rats could be a more valuable animal model for DD than *Lamp2*/KO mice	*RGD #13703119*
**Nervous system**	Epileptic encephalopathy, early infantile, 63 (EIEE63)	*Cplx1/KO*	CRISPR-Cas9	Xu et al., 2020	*Cplx1*/KO rats and mice show different phenotypes Rat model reproduces the disease better	
	Dystonia 25 (DYT25)	*Gnal/KO*	CRISPR-Cas9	Yu-Taeger et al., 2020	*Gnal/KO* rats show early symptoms as in patients not seen in*Gnal*/KO mice	/
	Cockayne syndrome	*Ercc6/KO (KI R571X)*	CRISPR-Cas9	Xu et al., 2019	The brain is more affected in CSB-deficient rats vs. mice	/
	Neonatal hydrocephalus	*L1cam/KO*	CRISPR-Cas9	Emmert et al., 2019b	*L1cam/*KO rats and mice show similar phenotypes similar to those of patients	*RRRC #850 + 851*
		*Ccdc39/KI point mutation c.916+2T*	CRISPR-Cas9	Emmert et al., 2019a	*Ccdc39*KO rats and mice show similar phenotypes Rats are more suitable for imaging and surgical experiments	/
	Schizophrenia	*Drd2/KI reporter*	CRISPR-Cas9	Yu et al., 2016	Inter-species difference of DRD2 expression between rats and mice	/
	Amyotrophic lateral sclerosis	*Fus/KI point mutation R521C*	CRISPR-Cas9	Zhang T. et al., 2018	Fus/KI rats and mice show an altered phenotype with subtle differences	/
	Neurofibromatosis type 1	*Nf1/KO*	CRISPR-Cas9	Moutal et al., 2017; Dischinger et al., 2018	*Nf1*/KO rats have a more pronounced phenotype than *Nf/* KO mice	/
	Cystic leukoencephalopathy	*RNaseT2/KO BigDel*	CRISPR-Cas9	Sinkevicius et al., 2018	No *RNaseT2*/KO mice reported	RGD #13781890, not available
	Epileptic encephalopathy, early infantile, 24 (EIEE24)	*Hcn1/KO*	TALEN	Nishitani et al., 2019	*Hcn1*/KO rats but not *Hcn1*/KO mice exhibited epilepsy	*NBRP Rat #0821 #0820 #0819 #0822*
	*MECP2*-related severe neonatal encephalopathy, Rett-like syndrome (RTT)	*Mecp2/KO*	ZFN	Engineer et al., 2015	*Mecp2/KO* rats displayed more symptoms of RTT than KO mice	*RGD #11567272; Horizon Discovery*
	Fragile X syndrome/Asperger syndrome, X-linked, 1 (ASPGX1)	*Fmr1/Nlgn3/DKO*	ZFN	Hamilton et al., 2014	Similar phenotype for Fmr1/Nlgn3/DKO rats and mice. Rats more suitable than mice for analysis of complex behavioral and social activities	RGD #11568700; Horizon Discovery; Nlgn3) RGD #11568040; Horzon Discovery; Fmr1 KO; RGD #11553873
	Phelan-McDermid syndrome	*Shank3/KO Shank3/KO BigDel*	ZFN CRISPR-Cas9	Harony-Nicolas et al., 2017 Song et al., 2019	*Shank3*-KO rats showed normal social interaction and self-grooming behaviors whereas *Shank3*-KO mice do not	/
	Angelman syndrome	*Ube3A/KO BigDel*	CRISPR-Cas9	Dodge et al., 2020	As in patients, *Ube3A/KO rats*bear a large deletion of the gene whereas*Ube3A/KO*mice not	/
	Intellectual deficiency from genetic origin	*Cplx1/KO*	CRISPR-Cas9	Xu et al., 2020	*Cplx1/KO* rats showed ataxia, dystonia, exploratory deficits, anxiety and sensory deficits but normal cognitive function	/
	Essential tremor	*Aspa and Hcn1/KO*	TALEN	Nishitani et al., 2020	*Aspa and Hcn1/KO* rats developed tremor	*NBRP Rat #0806 #0805 (Aspa KO)*; Cf Table 6 *pour Hcn1 KO*
	Ataxia-telangiectasia	*Atm/KO*	ZFN	Quek et al., 2017	*Atm*/KO rats show cerebellar atrophy and neurodegeneration which are poorly recapitulated in *Atm*/KO mice	*NBRP #0627 #0649*
	Autism spectrum disorder	*Cntnap2/KO*	ZFN CRISPR	Scott et al., 2018	*Cntnap2/KO* rats better recapitulate certain behavioral symptoms than*do Cntnap2*/KO mice	*RGD #11568646; Horizon Discovery; RGD #25330087 (CRISPR);*
		*Shank2/KO*	ZFN	Modi et al., 2018	*Shank2/KO* rats show behavior and electroencephalography abnormalities not seen in*Shank2*/KO mice	/
	Canavan disease	*Aspa/KO*	TALEN	Nishitani et al., 2016	*Aspa*/KO rats and mice show similar phenotypes similar to those of patients	*NBRP Rat #0806 #0805*
	Familial focal epilepsy	*Depdc5/KO*	TALEN	Marsan et al., 2016	Homozygous Depdc5/KO rats and mice have similar phenotypes but heterozygous Depdc5/KO rats and not mice had altered neuron excitability and firing patterns	NBRP Rat #0739
	Parkinson’s disease	*Lrrk2/KO*	ZFN	Ness et al., 2013	*LrrK2*/KO rats and mice show similar phenotypes similar to those of patients	*RGD #7241053; Lrrk1/Lrrk2 KO Horizon Discovery RGD #7241047; Lrrk1/Lrrk2 KO Horizon Discovery RGD #7241050; Lrrk2/KO; Horizon discovery RGD #7241056; Lrrk2/KO; Horizon Discovery*
	Alpha-synuclein autosomal dominants forms of Parkinson’s disease	*SNCA-A53T-A30P/Tg*	DNA microinjection	Lelan et al., 2011	*SNCA-A53T* transgenic rats and mice have similar phenotypes	/
	Familial Parkinson’s disease	*DJ-1 and Pink1/KO*	ZFNs	Sun et al., 2013	*DJ-1 and Pink1/KO* rats and mice show similar phenotypes similar to those of patients	*DJ-1 RGD #7241054 + RGD #7241049 Pink1/KO; Horizon discovery*
	congenital generalized lipodystrophy	*Bscl2/KO*	ENU	Ebihara et al., 2015	*Bscl2/KO* rats have brain reduction and azoospermia as in patients, *Bscl2/KO* mice do not reproduce these pathologies	*NBRP Rat #0763*
	Autosomal-dominant lateral temporal lobe epilepsy	*LGI1/KO*	ENU	Baulac et al., 2012	Rats reproduce the human disease and are complementary to the KO mice	NBRP Rat #0656
**Gastrointestinal**	Hereditary tyrosinemia type I	*Fah/KO*	CRISPR	Zhang et al., 2016	*Fah/KO* rats developed liver fibrosis and cirrhosis, not observed in Fah/KO mutant mice	*RGD #10002791 (TALEN; PhysGenKO) RGD #14398825 (CRISPR) RGD #14398828 (CRISPR*
	Hirschsprung disease	*Ednrb/KO*	CRISPR-Cas9	Wang et al., 2019a	*Ednrb*/KO rats in a particular strain caused embryonic lethality and megacolon as in certain strains of *Ednrb*/KO mice	/
	Rotor syndrome	*OATP1B2/KO*	CRISPR-Cas9	Ma et al., 2020	*OATP1B2/KO* rats reproduce the hyperbilirubinemia observed in patients	/
	Atypical hereditary non-polyposis colorectal cancer	*Msh6/KO*	ENU mutagenesis	van Boxtel et al., 2008	*Msh6/KO* develop a spectrum of tumors	/
	familial colon cancer	*Apc/KO*	ENU mutagenesis	Amos-Landgraf et al., 2007	Apc/KO recapitulates pathology better than mouse models	RRRC#00782 + RRRC#718 (Amos-Landgraf) NBRP Rat #0443
**Muscle**	Muscular dystrophy (Duchenne and Becker forms)	*Dmd/KO and BigDel*	TALENs and CRISPR-Cas9	Larcher et al., 2014; Nakamura et al., 2014	*Dmd/KO*rats better recapitulate the pathology than *Dmd*/KO mice	*NBRP Rat #0779 NBRP Rat #0780 NBRP Rat #0781 RGD #12880037; (TRIP)*
	Myostatin-related muscle hypertrophy	*Mstn/KO*	ZFN	Mendias et al., 2015; Gu et al., 2016	In contrast to Mstn/KO mice, Mstn/KO rats showed higher muscle fiber contractibility and lifelong increase in weight in male but not female	RGD #5131964 (PhysGen KO) RGD #5143985 (PhysGenKO) RGD #5131954 (PhysGen KO)
**Lung**	Cystic fibrosis	*Cftr/KO*	ZFN	Tuggle et al., 2014	*Cftr*/KO rat and mice show similar phenotypes that are mostly similar to those in patients. Rats but not mice have tracheal and bronchial submucosal glands.	*RGD #14392817 (SAGE, not available) RGD #14392813; Horizon discovery RGD #14392815; Horizon discovery*
		*Cftr/KO and DF508*	CRISPR-Cas9	Dreano et al., 2019	*Cftr*/KO and *DF508*rats and mice show similar phenotypes. *DF508*rats and mice show phenotypes that are milder than in their *Cftr*/KO counterparts. Rats but not mice have tracheal and bronchial submucosal glands	/
		*CFTR/KI and G5551D*	ZFN	Birket et al., 2020	CFTR/KI G5551D humanized rats display normalization of several pulmonary parameters after ivacaftor treatment	/
**Endocrine**	Glucocorticoid resistance	*Nr3c1/cKO*	CRISPR-Cas9	Scheimann et al., 2019	*Nr3c1/cKO* in CNS specific brain regions using injection of AAV-Cre vectors not possible in mice	/
	Estrogen resistance (ESTRR)	*Esr1/KO*and*Esr2/KO*	ZFN	Rumi et al., 2014; Khristi et al., 2019	*Esr1/KO*rats and mice show similar phenotypes similar to those of patients	*RRRC#701 (Esr1 KO) RRRC#849 (Esr1 KO) RRRC#742 (Esr2 KO) RRRC#677 (Esr2 KO)*
	Congenital hypothyroidism	*Tshr/KO*	CRISPR-Cas9	Yang et al., 2018	*Tshr/*KO rats and certain strains of*Tshr*KO mice show similar phenotypes similar to those of patients	/
	Allan-Herndon Dudley-syndrome	*Mct8/KO*	CRISPR-Cas9	Bae et al., 2020	*Mct8/KO* rats showed growth and reduced sperm motility and viability *Mct8/KO* mice did not show growth retardation	/
**Metabolic**	Congenital leptin deficiency	*Lep/KO*	CRISPR-Cas9	Guan et al., 2017	*Lep*/KO rats and mice show similar phenotypes similar to those of patients	/
	Leptin receptor deficiency	*Lepr/KO*	CRISPR-Cas9 and TALEN	Bao et al., 2015; Chen Y. et al., 2017	*Lep*/KO rats and mice show similar phenotypes similar to those of patients	/
	Aceruloplasminemia	*Cp/KO*	CRISPR-Cas9	Kenawi et al., 2019	*Cp*/KO rats show similar plasma biochemical alterations and profile of iron overload in liver and spleen as in humans *Cp*/KO mice showed different results	*RGD #38501060 #38501061 #38501059; not available*
	Multiple mitochondrial dysfunctions syndrome, among them pulmonary artery hypertension	*Nfu1/KI point mutation G206C*	CRISPR-Cas9	Niihori et al., 2020	*Nfu1/KI point mutation G206C* is only reported in rats. The model shows both mitochondrial dysfunction, and pulmonary artery hypertension with more prevalence in females than in males, as in patients	/
	Generalized arterial calcification of infancy and pseudoxanthoma elasticum	*Abcc6/KO*	ZFN	Li et al., 2017	*Abcc6/KO* rats allowed ex vivo perfusion of liver and spleen and definition of the liver as the primary site of the disease	*RGD #13792683 #13792682 #10413850 #10413852 #10413854 #10413858 #10413856*
	Diabetes mellitus, non-insulin-dependent, 5 (NIDDM5)	*AS160 (TBC1D4)/KO*	CRISPR-Cas9	Arias et al., 2019	*AS160-KO*rats and mice showed similar alterations in whole body assessment Rats’ bigger size allowed measurements using single myofibers	*RGD #38596327*
	multiple mitochondrial dysfunctions syndrome	Isca1/KI-mCherry-Cre	CRISPR-Cas9	Yang et al., 2019	Developmental block in embryos at 8.5 days Not reported in mice	/
	Primary hyperoxaluria type 1 (PH1)	*Agxt/KO*	CRISPR-Cas9	Zheng et al., 2020	*Agxt*/KO rat model better recapitulate the disease than the *Agxt/KO*mice	/
		*Agxt/KI mutation D205N*	CRISPR-Cas9	Zheng et al., 2018	*Agxt/KI mutation D205N* model recapitulates the disease in rats Not reported in mice	/
	Familial hypercholesterolemia	*Ldlr-ApoE/DKO*	CRISPR-Cas9 and CRISPR-Cpf1	Zhao et al., 2018; Lee J. G. et al., 2019	Double*Ldlr-ApoE/DKO* rats better recapitulate the pathology than do double*Ldlr-ApoE/DKO* mice	/
	Dwarfism	*Ghsr/Tg Ghsr/KO*	DNA microinjection ENU mutagenesis	Flavell et al., 1996 Shuto et al., 2002	Dwarfism in rats as in *GshR*/KO mice Analysis of the role of GSHR in behavioral pathologies including eating disorders	RGD #12910127 RGD #1642278 (PhysGen) RRRC#421RRRC #405
		*Ghsr/KO*	CRISPR-Cas9	Zallar et al., 2019		RRRC#827
	Hyaline fibromatosis syndrome	*Antxr2/KO*	CRISPR-Cas9	Liu X. et al., 2017	*Antxr2*/KO rats and mice show similar phenotype *Antxr2*/KO rats did not develop hypertension	/
	Obesity (OBESITY)	*Mc3R-Mc4R/DKO*	CRISPR-Cas9	You et al., 2016	Double*Mc3R-Mc4R/DKO* rats better recapitulate the pathology than do double*Mc3R-Mc4R/DKO* mice	RGD #13825199 (Mc4R KO) (Hubrecht Laboratory, Centre for Biomedical Genetics, 3584 CT Utrecht, The Netherlands. Hera Biolabs, Taconic.)
	Congenital hyperinsulinism	*Sur1/KO*	TALEN	Zhou et al., 2019	*Sur1*/KO rats and mice reproduce the disease Rats showed a particular glucose control profile	/
	Fumarase deficiency	*Fh/KO*	TALEN	Yu et al., 2019	*Fh*/KO rats and mice show similar phenotype and reproduce the disease	*RGD #13792795 #13792794 (not available)*
	Fabry disease	*Gla/KO*	CRISPR-Cas9	Miller et al., 2018	*Gla/KO* rats better recapitulate the pathology than do *Gla/KO* mice	*RGD #10054398*
	Oculocutaneous albinism type 1	*Tyr/KO*	TALEN	Mashimo et al., 2013	*Tyr*/KO rats and mice show similar phenotype and reproduce the disease	*NBRP Rat #0666*
	Wolfram syndrome	*Wfs1/KO*	ZFN	Plaas et al., 2017	*Wfs1/KO*rats better recapitulate the pathology than *Wfs1/KO* mice	/
**Nephrology**	Focal segmental glomerulosclerosis 2 (FSGS2)	*Trpc6/KO BigDel*	CRISPR-Cas9	Kim E. Y. et al., 2018	*Trpc6*/KO rats and mice were protected from FSGS2	*RGD #11553908 #11553912 #11553902*
	C3 glomerulopathy	*C3/KO C3/KO*	ZFN CRISPR-Cas9	Negishi et al., 2018) Xu et al., 2018	C3/KO rats and mice display a similar phenotype Most mouse strains have a defective complement system downstream of C3	/ RGD #19165133
	REN-related kidney disease	*Ren/KO*	ZFN	Moreno et al., 2011	Rats like humans have 1 copy of the Ren gene whereas mice have 2 copies Rats faithfully recapitulate the disease	RGD #4139880 (PhysGen)
**Ophthalmology**	Autosomal dominant congenital stationary night blindness and retinitis pigmentosa	*Rho s334ter/Tg*	DNA microinjection	Liu et al., 1999	This is a unique widely used model of this disease	
	Retinitis pigmentosa 85 (RP85)	*Ahr/KO*	ZFN	Harrill et al., 2013	Ahr/KO rats and mice showed distinct phenotypes in the eye, liver and kidneys during normal development and toxic responses	Cf Table 6
	Autosomal dominant congenital stationary night blindness	*Pde6b/KO*	CRISPR-Cpf1	Yeo et al., 2019	*Pde6b* /KO rats and mice reproduce the disease Slower progression and larger anatomic architecture in rats are advantages versus the mouse model	/
	Familial exudative vitreoretinopathy	*Lrp5/KO*	CRISPR-Cas9	Ubels et al., 2020	*Lrp5/KO*rats show retinal and bone abnormalities Similar phenotype in*Lrp5/KO*mice	/
**Cancer**	Li-Fraumeni syndrome	*Tp53*	ES ZFN	McCoy et al., 2013	*Tp53/KO* rats developed more diverse tumors and more frequently than *Tp53/KO* mice	*RGD #12904897 (Horizon Discovery) RGD #11553886NBRP Rat #0726 RRRC #00485 (ES)*
**Immune and hematological systems**	Von Willebrand disease	*Vwf/KO BigDel*	CRISPR-Cas9	Garcia et al., 2020	*Vwf*/KO rats and mice display a similar phenotype	*RGD #18182946 #39128242 #18182944*
	Hemophilia A	*F8/KO*	ZFN	Nielsen et al., 2014	*F8*/KO rats and mice show similar phenotype	*RGD #11531094 (Novo Nordisk, Maaloev, Denmark)*
		*F8/KO (gene inversion)*	CRISPR-Cas9	Shi et al., 2020		RGD #13800746
	ALSP	*Csf1r/KO*	ES cells	Pridans et al., 2018	*Csf1r/KO* rats showed a more severe phenotype than patients and *Csf1r/KO* mice an even stronger one	/
	SCID	*Rag1/KO*	Meganucleases and CRISPR-Cas9	Tsuchida et al., 2014; Zschemisch et al., 2012; Ménoret et al., 2013	*Rag1*/KO rats and mice show similar phenotype	Cf Table 6
		*Rag2/KO*	CRISPR-Cas9	Liu Q. et al., 2017; Noto et al., 2018	*Rag2*/KO rats and mice show similar phenotype	Cf Table 6
		*Prkdc/KO*	CRISPR-Cas9	Mashimo et al., 2012; Ma et al., 2014a	*Prkdc*/KO rats and mice show similar phenotype	Cf Table 6
	X-linked SCID	*Il2Rg/KO*	ZFN, TALEN and CRISPR-Cas9	Mashimo et al., 2012; Samata et al., 2015; Kuijk et al., 2016; Ménoret et al., 2018	*Il2rg*/KO rats and mice show similar phenotype	Cf Table 6
	APECED	*Aire/KO*	TALEN	Ossart et al., 2018	*Aire*/KO rats showed a more pronounced phenotype than *Aire*/KO mice	Cf Table 6
	Agammaglobulinemia non-Bruton type	*Ighm/KO*	TALEN CRISPR-Cas9	Ménoret et al., 2010; Panzer et al., 2018	*Ighm*/KO rats and mice show similar phenotype	Cf Table 6

### Strategies to Develop Genetically Modified Models

#### Single, Multiple or Large Modifications

A KO model can be efficiently generated through out-of-frame indels (Figure 4A) by careful design of gRNA. Some of these will lead to a reading frame shift with a premature termination codon followed by mRNA degradation and no translation of the protein. All mechanisms of premature termination codon followed by mRNA degradation are not fully understood on mammals and exceptions exist (Dyle et al., 2020). Most often, the CRISPR-Cas system is designed to target one of the first exons of the gene, but another approach is to generate a promoter-less allele that can lead to a more severe phenotype than the KO model (El-Brolosy et al., 2019). In that case, KO can be easily confirmed by detection at the mRNA level. This strategy has not been used commonly, but it could be particularly useful in the rat, for which protein detection tools are limited. Mainly, these models have been developed by nuclease DSB induction, but adenosine-base editor is also an alternative with mouse and rat (Ma Y. et al., 2018; Yang L. et al., 2018; Wang X. et al., 2020).

Multiple KO models can be generated using multiple RNP complexes (Ma et al., 2014a,b), but to avoid large deletions, they should not be located on the same chromosome (Figure 4A). Translocation between chromosomes is also a risk that can be reduced using ssODNs and different Cas (Bothmer et al., 2020). Outcomes analysis for multiple KO can be challenging and should be carefully considered when designing CRISPR tools.

For large genomic KOs involving several consecutive genes, two DSBs can be induced by designing gRNA on both sides of the region of interest (Figure 4A). If both DSBs occur at the same time, the result will be a large deletion of this region of interest. To our knowledge, the biggest deletion achieved to date in rats is 24,499 Kb (Birling et al., 2017).

ssODNs that include a STOP codon can be used to create a nonsense mutation and inactivate a specific gene (Figure 4B). The rate of KI is usually lower than the frequency of indels, but because both the KI and a large fraction (>70%) of indels (Guo et al., 2018; Taheri-Ghahfarokhi et al., 2018) induce out-of-frame mutations, this increases the chance of obtaining a KO animal.

ssODNs containing a mutation observed in a human disease have been used to generate animal models (Figure 4B) such as for cystic fibrosis (Dreano et al., 2019; Table 7). The use of ssODNs will allow inclusion of specific features, such as restriction sites, to facilitate KI genotyping. Base- and prime-editing, are particularly fitting tools for generating mutations. Base editing has already been applied in the rat (Yang L. et al., 2018) but prime editing only in the mouse for now (Liu Y. et al., 2020).

#### Gene Overexpression

Overexpression of the gene of interest might be useful for gaining a better understanding of its role. The gene can be overexpressed by its insertion with its promoter or with an ubiquitous promoter (Figure 4C, right panel). In the past, this effect has been achieved through transgenesis, but expression of a randomly inserted cassette is affected by the genomic locus where it is inserted. Advances in genome-editing tools have made it possible to target a permissive locus, also called a “safe harbor,” to overcome this issue (Saunders, 2020). *Rosa26* and *Hprt* are the most commonly used safe harbors that have been targeted in rat embryos (Kobayashi et al., 2012; Remy et al., 2014).

Humanized animal models are of great value to better study human diseases by insertion of the human gene into the animal genome (Figure 4C, right panel). For some projects, cDNA of the gene of interest is enough and can be used to generate humanized models, as it was done for a humanized model of cystic fibrosis (Birket et al., 2020).

#### Conditional Models

Site-specific recombinase systems (SSR) are used for conditional excision or inversion of the targeted site. Their application requires the generation of two lines, one expressing the specific SSR and one displaying the two specific DNA sites flanking the locus of interest (Figure 4C, lower panel). These lines are then crossed to combine both mutations in a single animal line (Birling et al., 2009). The Cre/lox system is the most commonly used SSR system option for mouse conditional models, even though other variants and other systems (FLP-FRT, Dre-rox, Nigri-nox, and others) have been used and combined. To the best of our knowledge, Cre/lox is the only SSR system that has been used to generate conditional rat models. The use of targeted nucleases permits precise insertion of Cre behind the endogenous promoter (Figure 4C, lower panel), allowing reliable and relevant tissue or cell specific expression of Cre (for a review see Kim H. et al., 2018). To achieve temporal control of the gene of interest, drug-inducible systems are used (Navabpour et al., 2020). Fusion of Cre with estrogen receptor 2 (Cre-ERT2) leads to sequestration of Cre in the cytoplasm, and the addition of tamoxifen at a certain time point induces Cre-ERT2 translocation into the nucleus, allowing Cre to recombine loxP sites (Figure 4C, lower panel). These animal lines should be carefully bred and analyzed to limit toxicity and leakage (Song and Palmiter, 2018). Cre/CreERT2 models characterization at some point requires the use of Cre reporter models expressing a floxed STOP before a reporter gene (Figure 4C, lower panel). After Cre recombination, reporter expression is turned on and specific expression can be characterized. Validation of loxP models requires Cre or CreERT2 models (Figure 4C, lower panel). The observed phenotype will then be specific to the Cre expressing tissues and the loxP line tested.

Other systems have been used in mouse and rat for spatiotemporal control. Tetracycline (Tet) on or off systems, like SSR systems, require two lines, one carrying a Tet (or doxycycline, its derivative)-sensitive transcriptional activator and one on the targeted locus carrying the Tet-responsive promoter element (Kim H. et al., 2018). The use of Tet systems for the development of transgenic mice has been reviewed previously (Sun et al., 2007) and applied to the generation of inducible rat models (Tesson et al., 1999; Table 6). For cell specific depletion, the diphtheria toxin receptor can be expressed under a cell specific promoter such as CX3CR1 for microglia depletion in rat (Vichaya et al., 2020).

Rat research is long way behind mouse studies for development of conditional models because of the decades-long use of mouse ES cells (Ramírez-Solis et al., 1995). Use of ES cells remains time consuming in mouse and technically challenging in rat. Efforts have currently been deployed to generate conditional models using CRISPR-Cas9 with all the difficulties previously discussed for large and complex insertion. Overcoming these hurdles is a major issue for both mouse and rat but it is required for the rat. A multicenter study in mice showed that loxP KI using two ssODNs and RNP complexes is less efficient than using a single long DNA donor (Gurumurthy et al., 2019a). Sequential insertion of each loxP ssODN by microinjection and electroporation of one and two-cell embryos has also been tested but is technically demanding (Horii et al., 2017).

#### Reporter and Tagged Rat Models

Transgenic ubiquitous reporter models have been generated with different fluorophores and promoters. The most developed and used models are animals that express fluorogenic proteins in different tissues, such as CAG-GFP rats (Remy et al., 2014; Ménoret et al., 2015). Today, with CRISPR-Cas systems, a reporter gene or a tag can directly be inserted at the end of the reading frame by replacing the stop codon of the endogenous locus of interest (Figures 4B,C, upper left panel). A fusion protein or two separated molecules expressed at the same level can be generated using self-cleaving peptides. Our team has generated a KI IL22bp-T2A-eGFP rat model to identify cells expressing this gene (submitted). For advanced reporter models, conditional tools can be used and combined, in particular for genetic lineage tracing (Liu K. et al., 2020).

### Models to Study Genes of the Immune System

In general terms, rats share more immune characteristics with humans than mice do (Wildner, 2019). As an example, complement levels in humans and rats are comparable (Ong and Mattes, 1989; Ménoret et al., 2020), whereas in most inbred mouse strains, they are undetectable or very low because of different genetic mutations (Ong and Mattes, 1989; Wetsel et al., 1990; Shultz et al., 1995).

The roles of genes identified in different immune pathophysiological processes, as well as others involved in normal immune responses, also have been analyzed and are listed in Table 6. For the sake of space and relevance of the rat model, only some of these generated genetically modified models are described in more detail below.

#### Immunodeficient Rat Strains

KO of genes involved in early rearrangements of immunoglobulin in B cells and of the T cell receptor genes in T cells, such as *Rag1* (Zschemisch et al., 2012; Ménoret et al., 2013; Tsuchida et al., 2014), *Rag2* (Kuijk et al., 2016; Liu Q. et al., 2017; Noto et al., 2018), and *Prkdc* (Mashimo et al., 2012; Ma et al., 2014a; Beldick et al., 2018) have resulted in defective development of B and T cells (Tables 6, 7). KO of the gamma chain receptor of the IL-2 receptor (*Il2rg*) results in defects of differentiation of T, B, natural killer (NK), and innate lymphoid cells (Mashimo et al., 2010; Samata et al., 2015; Kuijk et al., 2016). Additionally, rat lines combining several genetic modifications, such as with the *Rag1*, *Rag2*, *Il2rg*, *Prkdc*, and *Foxn1* genes, have been developed (Mashimo et al., 2012; Goto et al., 2016; Ménoret et al., 2018; He et al., 2019). Transgenic rats for human SIRPa to inhibit phagocytosis in human cells have been described in recent years (Goto et al., 2016; Jung et al., 2016; Yang X. et al., 2018; Ménoret et al., 2020). These rats have been used in humanization of their immune system and/or other tissues in transplantation and regenerative medicine settings (for a review, see Adigbli et al., 2020) and in cancer research (He et al., 2019). In these models as in others, the larger size of the rat allows to do analysis of human cells of the blood more frequently than in mice. Furthermore, the normal complement levels in rats allow to analyze the effector function of different anti-human antibodies, not possible to do in mice (Ménoret et al., 2020). Other genetic modifications to improve immune or liver humanization that have been developed in mice, will probably also be applied to the present generation of immunodeficient rats (Adigbli et al., 2020).

B cell–deficient rats have been described (Ménoret et al., 2010; Panzer et al., 2018) and used in organ transplantation models, and the rat may better recapitulate lesions mediated by complement activation through antibodies in the transplantation setting (Platt and Cascalho, 2018). One of these B cell–deficient strains (Ménoret et al., 2010) was obtained by disrupting the J sequence of the immunoglobulin heavy chain and further rendered deficient for both immunoglobulin light chains (Osborn et al., 2013). With the objective of generating fully human monoclonal antibodies (mAbs), these immunoglobulin-deficient rats were humanized for immunoglobulins by transgenesis using BACs (Osborn et al., 2013). These animals can generate human mAbs with diversity and affinity (Osborn et al., 2013) and different versions of these animals have been generated (Harris et al., 2018; Clarke et al., 2019).

Inactivation of the C3 complement gene has allowed confirmation of a new role for complement in a model of polyneuropathy following chemotherapy. As stated earlier, the fact that complement levels in humans and rats are comparable (Ong and Mattes, 1989; Ménoret et al., 2020), makes the rat a model of choice for exploring the role of complement in different pathological situations (Xu et al., 2018).

#### Cluster of Differentiation (CD) or Other Cell Membrane Molecules

In model of neuromyelitis optica induced by passive administration of human IgG autoantibodies targeting aquaporin-4, rats deficient in the cell membrane inhibitor of complement activation CD59 showed a much more pronounced neurological pathology than CD59 KO mice (Yao and Verkman, 2017a,b). This model emphasizes the role of complement in this pathology and the availability of a more relevant model of the disease than mice.

CLEC-1 is a cell membrane receptor expressed by dendritic cells (DCs) that reduces immune responses and plays a role in immune tolerance models (Thebault et al., 2009). CLEC-1 KO rats show enhanced *Il12p40* subunit mRNA expression in DCs and an exacerbation of downstream *in vitro* and *in vivo* CD4^+^ Th1 and Th17 responses (Lopez Robles et al., 2017).

Human and rat (Maruoka et al., 2004) but not mouse cells express the Fc receptor for IgA (FcaRI, CD89; mice bear only a *FcarI* pseudogene) (Launay et al., 2000). CD89 KO rats have been generated and have provided interesting new information on a model of IgA-induced nephropathy a frequent pathology in humans (submitted).

Similarly, human and rat DCs display quite similar profiles of Toll-like receptor (TLR) expression in different DC subsets, allowing to better explore their role in infectious and inflammatory diseases. DCs from both species express the TLR10, whereas mouse DC subsets do not show a particular profile of TLR expression and TLR10 is not expressed (mice bear only a *Tlr10* pseudogene) (Hubert et al., 2006). Rats deficient for TLR10 have been generated and are being characterized (in preparation).

A human CD4/CCR5 transgenic rat model (Keppler et al., 2002) has been extensively used to analyze different aspects of HIV infection and treatment with more relevant results as compared to mice with similar transgenes (Goffinet et al., 2007).

In humans, HLA-B27 is strongly associated with a series of inflammatory diseases grouped together under the term “spondyloarthropathies.” In contrast to the negative results in transgenic mice, transgenic HLA-B27 rats spontaneously develop inflammatory disease in the same organs as those involved in humans (Hammer et al., 1990). This model has been extensively used and is the model of choice in this pathology (for a review, see Braem and Lories, 2012).

#### Cytokines and Their Receptors

*Il22bp* KO rats show that IL22-binding protein is protective in models of inflammatory colitis (Martin et al., 2016) and psoriasis (Martin et al., 2017). *Il22bp*-GFP KI rats have facilitated precise definition of cell subsets that express IL22bp by different subsets of DCs in different tissues (submitted).

Viral infections can trigger autoimmune diabetes in rats and type I IFN α/β receptor (IFNAR1) KO rats have a significantly delayed onset and frequency of diabetes. These findings support the idea that innate immunity influences autoimmune diabetes and encourage the use of targeted strategies to inhibit type I IFN α/β (Qaisar et al., 2017).

NK cells could play a role in placenta generation, and IL-15 KO rats showed an absence of NK cells and several abnormal placental characteristics, supporting a role for NK cells (Renaud et al., 2017).

A *Csf1r* reporter gene (Irvine et al., 2020) and *Csf1r KO* (Pridans et al., 2018) lines are useful tools for the analysis of macrophages and of CSF1R biology (Hume et al., 2020). CSF1R is also the receptor for IL-34, and *Il34*-mutated rats exhibit depletion of microglia and Langerhans cells, as well as defects in tolerogenic immune responses (submitted).

#### Intracellular Molecules

Certain molecules that regulate metabolic functions in many cell types, including in immune cells, have been analyzed using genetically modified rats. Transgenic rats for heme oxygenase-1 (HO-1) under the control of the ubiquitous H-2Kb promoter (Braudeau et al., 2003) and HO-1 KO rats (Atsaves et al., 2017) have facilitated dissection of different aspects of HO-1 effects, particularly in kidney, where the lesions observed in rats differ from those in mice.

The hydrocarbon receptor (AHR) is a transcription factor with an essential role in mediating toxic responses to environmental pollutants and in regulating many cellular pathways involving endogenous ligands. In *Ahr* KO rats, the percentages of T CD3+, T CD8+, and CD11c+ cells in the spleen and the activation of T cells are decreased, whereas the percentage of NK T cells and the activation of B cells is increased compared to wild-type rats (Phadnis-Moghe et al., 2016).

The lymphopenia observed in diabetic biobreeding rats results from a spontaneous mutation in the immune-associated nucleotide gene 5 (*Ian5*), a protein expressed in the mitochondria membrane where it regulates apoptosis. Lymphocyte numbers are normalized when a normal *Ian5* gene is transgenically expressed (Michalkiewicz et al., 2004).

Some of the most commonly used immune system models developed in rats are based on intrinsic characteristics of the species. For example, the rat has always been an important model of autoimmune arthritis (Holmdahl et al., 2001) and HLA-B27 transgenic rats recapitulate spondyloarthropathies much better than do HLA-B27 transgenic mice.

Certain immune reagents, such as antibodies recognizing leukocyte differentiation antigens, are less abundant in rats than in mice but more so than in other experimental species. High-density flow cytometry techniques have not yet been applied in the analysis of the rat immune system and will clearly be of great interest when coupled with modification of rat immune system genes.

### Genetic Diseases Models

For 150 years, spontaneous or induced (ENU) genetic mutations in the rat have been used as models of human genetic diseases. For a decade, the advent of genetic engineering tools such as ZFN, TALEN, and CRISPR-Cas have led to a real revolution in obtaining specific and targeted genetic mutations in rats for the study of human genetic diseases. These advances, coupled with historical knowledge and use of the rat in many research fields, have increased the generation of rat models of human genetic diseases. More than 6000 genetic diseases have been described, and several databases have recorded variants that are associated with or responsible for genetic diseases. Several important genetic diseases have been modeled in rats. A complete list is presented in Table 7, and a brief description of the most useful models is provided below.

#### Cardiovascular Diseases (CVD)

Because of its larger size allowing catheterization, lower cardiac frequency versus mice, and historical use in CVD, the rat has been an important model for a series of genetically modified rat models of CVD.

Pulmonary arterial hypertension (PAH) results from a reshaping and thickening of the walls of medium and small caliber pulmonary vessels. By their frequencies and effects, the mutations in the BMPR2 gene are the main variants responsible for inheritable forms of isolated PAH. *Bmpr2* KO rats show some of the critical clinical, cellular, and molecular dysfunctions described in human PAH both in the heart and vessels (Ranchoux et al., 2015; Hautefort et al., 2019; Manaud et al., 2020). Although rarer, mutations in the KCNK3 gene encoding a potassium channel have also been described as causative in PAH. *Kcnk3* KO rats develop age-dependent PAH associated with characteristic electrophysiological and molecular alterations in the myocardium and vessels (Lambert et al., 2019). Because the *Kcnk3* gene is not functional in mice, this rat model offers new insights into the mechanisms of PAH and in the testing of therapeutics.

To investigate the role of the MYL4 gene in atrial cardiomyopathy, Myl4-KO or mutated rats have been generated. Both show a phenotype similar to affected patients and are new models for further mechanistic analysis (Peng et al., 2017).

Danon disease (DD) is a metabolic disease caused by mutations in the LAMP2 gene, and the most common symptom is cardiomyopathy. Recently generated *Lamp2* KO rats show similarities to DD patients at the heart tissue level and with multisystem lesions, constituting an important new animal model of DD (Ma S. et al., 2018).

#### Neurological Diseases

In neurobiology and cognitive studies, the rat, because of its larger size and more complex and richer behavior, is preferred as a rodent model. Genetically modified rats have provided several important models for neurological disorders with a genetic component.

Mutations in complexin-1 (CPLX1) gene lead to epileptic encephalopathy with onset on infancy. *Cplx1* KO rats have different phenotypes from mice. Both show profound ataxia, but in rats, behavior is more affected, and they have more abnormal histomorphology of the stomach and intestine, resulting in early death (Xu et al., 2020).

A nonsense mutation in the Cockayne syndrome B gene, *Ercc6*, more profoundly affects the rat brain than the mouse KO for the same gene (Xu et al., 2019). In these rats, RNA-seq analysis has revealed transcription dysregulation that contributes to the neurologic disease.

Neonatal hydrocephalus has been analyzed using two different models of mutated rats, one with an invalidation of the *L1cam* gene (Emmert et al., 2019b) and the other with a KI of a specific mutation in the *Ccdc39* gene (Emmert et al., 2019a). These models allow for neurosurgery procedures that are difficult to perform in mice, with resulting characterization of the lymphatic-mediated cerebrospinal fluid circulation and inflammation in this disease.

As a model for familial amyotrophic lateral sclerosis, rats with a FUS point mutation KI via CRISPR-Cas9 express a physiological level of this mutant, along with cognitive impairment and neuromuscular signs. In this rat model, FUS KI highlighted sleep–wake and circadian disturbances as early alarm signals (Zhang T. et al., 2018).

Neurofibromatosis type 1 is an autosomal dominant disease arising from mutations in the NF1 gene that results in the development of tumors in the nervous system, neurological disorders and chronic idiopathic pain (Dischinger et al., 2018). *Nf1* KO rats show increased nociceptor excitability and hyperalgesia. These models are important in the search for a potential key target (CRMP2) for therapeutic intervention (Moutal et al., 2017).

RNASET2 deficiency in humans is associated with cystic leukoencephalopathy. *RnaseT2* KO rats are the only rodent model of this disease. Despite a less severe neurodegeneration phenotype than in patients, this model is useful for studying RNASET2 function, especially for hippocampal neuroinflammation (Sinkevicius et al., 2018).

A group of neurodevelopmental diseases, gathered under the name of autism spectrum disorders (ASDs), are characterized by heterogeneous capabilities in social interactions and by stereotyped behaviors. One subtype of ASD is associated with mutations in the MECP2 gene, causing an X-linked neurodevelopmental disorder named Rett syndrome. *Mecp2* KO rats clearly show both motor and behavioral deficits early in development, more pronounced than in mice (Patterson et al., 2016). Another subtype of ASD is ASD/Fragile X syndrome. Two KO rat models have been generated for this condition, one syndromic (*Fmr1*) and one non-syndromic (*Ngln3*) (Hamilton et al., 2014). These KO rats show some ASD-relevant phenotypes for investigations at the genetic level. Phelan–McDermid syndrome is another ASD-associated condition, caused by mutations in the SHANK3 gene. In contrast to *Shank3* KO mice, *Shank3* KO rats showed normal social interaction but impaired social memory (Harony-Nicolas et al., 2017; Song et al., 2019). Similarly, *Shank2* KO rats better recapitulate the condition than the KO mice (Modi et al., 2018). Angelman syndrome results from mutations in the *UBE3A* gene, which in most cases is a large gene deletion, and in a small fraction with mutations in exon 2. The *Ube3A* mouse model bears a null mutation of exon 2, whereas the rat model is closer to the human condition with a large deletion of the *Ube3a* gene. The rat model mimics human Angelman syndrome with abnormalities in motor coordination and cognitive function (Dodge et al., 2020).

#### Muscular Diseases

Myopathies are a set of neuromuscular diseases, the most common of which is Duchenne’s muscular dystrophy (1 in 3300 newborn babies) resulting from mutations in the dystrophin gene (DMD). As in humans, *Dmd* KO rats show decreased muscle strength as well as a degradation/regeneration phenotype in skeletal muscles, heart, and diaphragm (Larcher et al., 2014; Nakamura et al., 2014). Of note, *Dmd* KO rats but not mice present cardiovascular alterations close to those observed in humans, which are the main cause of death in patients. All of these clinical signs and pathological features are much more pronounced than in *Dmd* KO mice. Rats are becoming an increasingly used model for the study of different aspects of Duchenne’s and Becker’s myopathies, including biomarkers, neurological abnormalities, and immune/inflammatory responses (Robertson et al., 2017; Ouisse et al., 2019; Caudal et al., 2020; Szabó et al., 2021).

#### Pulmonary Diseases

Cystic fibrosis is one of the most common genetic diseases in western populations (approximately 1 in 4000 newborns) and is caused by mutations in the *CFTR* gene. The most common mutation in humans is the missense mutation DF508, which leads to abnormal CFTR function and mucus accumulation. Cystic fibrosis is characterized by airway and digestive pathology with a reduced life expectancy. Mice do not have submucosal glands, in contrast to humans and rats. Rats with the DF508 mutation (Dreano et al., 2019), as well as with a complete KO for *Cftr*, have been generated (Tuggle et al., 2014; Dreano et al., 2019). *Cftr* KO rats showed a very severe digestive phenotype and lung lesions in surviving older animals, and reduced weight and life expectancy, although milder in DF508 rats. Very recently, a humanized model of cystic fibrosis was created by inserting the human CFTR cDNA sequence harboring a G551D mutation by KI into the rat genome, downstream of the endogenous *Cftr* promoter (Birket et al., 2020).

#### Metabolic Diseases

To study disorders of metabolism, leptin, a cytokine-like hormone principally produced by white adipose tissues, was deleted in rats. Microarray analysis has been performed in *Lep* KO rats to evaluate alterations in white adipose gene expression and to explore pathways involved in metabolic diseases with leptin deficiency (Guan et al., 2017). The leptin receptor (*Lepr*) has also been deleted in rats, and these animals show hyperphagia, obesity, hyperglycemia, and dyslipidemia. This model could complement the existing models (db/db mice and Zucker rats) and be useful for research in obesity and diabetes (Bao et al., 2015; Chen Y. et al., 2017).

Hereditary aceruloplasminemia is a genetic disease characterized by progressive iron overload (liver and brain) and is related to mutations in the ceruloplasmin (*CP*) gene. In contrast to *Cp* KO mice, *Cp* KO rats mimic the human phenotype with hepatosplenic iron load and could be more appropriate for providing information to understand and treat the disease (Kenawi et al., 2019).

Abnormal calcification and phosphate deposition are the basis of generalized arterial calcification of infancy and pseudoxanthoma elasticum, both caused by mutations in the *ABCC6* gene. These mutations lead to generalized arterial calcification through the body in infancy. Because ABCC6 is expressed in liver and kidney, an important question is the respective role of these organs in the generalized disease. Given their small size, mice KO for *Abcc6* are not suitable for *ex vivo* perfusion experiments. *Ex vivo* perfusion of liver and kidneys from *Abcc6 KO* rats has revealed that the liver is the primary site of molecular pathology in these process and points to a preferential target of the liver to treat them (Li et al., 2017).

The low-density lipoprotein receptor (LDLR) and apolipoprotein E (APOE) genes control normal levels of cholesterol and other forms of fat in the blood. A deficiency in LDLR is the cause of familial hypercholesterolemia and a deficiency in APOE is involved in several age-related fatty acid diseases. Recently, two reports (Zhao et al., 2018; Lee J. G. et al., 2019) described double-KO for *Ldlr* and *Apoe* genes in rats. These rats mimic more closely than KO mice the pathological changes observed in hyperlipidemia and atherosclerosis in humans with genetic deficiencies and in normal individuals.

Melanocortin-3 and -4 receptors (MC3R and MC4R) regulate energy and body weight. *Mc3R-Mc4R* double-KO rats exhibit worse phenotypic features than single-KO rats and *Mc3R-Mc4R* double-KO mice (You et al., 2016).

Fabry disease is an X-linked lysosomal storage disease caused by α-galactosidase A (α-Gal A) deficiency resulting from mutations in the GLA gene. *α-Gal A* KO mouse models do not recapitulate the cardiorenal findings observed in humans and *Gla* KO rats more closely mimic the disease phenotypes observed in patients (Miller et al., 2018).

Wolfram syndrome (WS) is a genetic disorder caused by mutations in the *WFS1* gene. Previous mouse models of WS involved only partial diabetes and other symptoms of the disease, whereas *Wfs1* KO rats developed diabetes as well as neuronal degeneration, as do patients (Plaas et al., 2017).

#### Kidney Diseases

Renin (REN) mutations are involved in REN-related kidney disease and tubular dysgenesis. The role of RAS in the regulation of blood pressure and kidney function has been extensively analyzed in rats (Jacob, 2010), including the generation of one of the first transgenic rat models (Mullins et al., 1990). Although humans and rats have only one copy of the renin gene, mice have two genes and thus increased renin expression levels (10-fold higher than their one-copy counterparts) (Hansen et al., 2004). *Ren* KO rats have lower blood pressure and severe kidney underdevelopment, reproducing the kidney lesions observed in REN-related kidney disease and tubular dysgenesis (Moreno et al., 2011).

#### Ophthalmology Diseases

Retinitis pigmentosa (RP) is a group of inherited mutations causing photoreceptor degeneration, loss of night vision, and blindness. Rhodopsin mutations comprise an important fraction of autosomal dominant RP. Transgenic rats harboring the *Rho s334ter* mutation are a widely used model for this pathology (Liu et al., 1999).

As noted, AHR is a ligand-activated transcription factor involved in the development of multiple tissues and activated by a large number of exogenous toxic compounds and endogenous ligands, such as kynurenines. *Ahr* KO rats and mice show ophthalmologic lesions as well as different renal and hepatic developmental and homeostatic lesions (Harrill et al., 2013).

#### Cancer

The tumor suppressor TP53 is a central player in cancer biology, and mutations in the TP53 gene are the most frequent mutations observed in human cancers. *Tp53* KO rats develop a wide variety of tumors, most frequently sarcomas, which are rarely observed in mice. These rats have been used in carcinogenicity assays for drug development (McCoy et al., 2013).

#### Immune and Hematological Systems

For hemophilia A, *FvIII* KO rats have no detectable FVIII activity, and their activated thromboplastin time and clotting time are significantly prolonged. Episodes of spontaneous bleeding requiring treatments were observed in 70% of the *FvIII* KO rats (Nielsen et al., 2014; Shi et al., 2020). In the rat genome, it is interesting to note that the *F8* gene is situated on chromosome 18, rather than the X chromosome as in humans, mice, dogs, and sheep (Lozier and Nichols, 2013).

Monocyte colony-stimulating factor (CSF-1) is, along with IL-34, a regulator of macrophages and myeloid DC development, acting through the CSF-1R (Ma et al., 2012). Humans with point mutations or less frequently deficiency for CSF-1R develop adult-onset leukoencephalopathy with axonal spheroids and pigmented glia, likely because of a decrease in the number of microglia (Hume et al., 2020). *Csf1r* KO rats (Pridans et al., 2018) develop some or all of the symptoms and lesions of the disease, but with greater severity and more bone lesions than in humans, whereas *Csf1r* KO mouse models show an even more severe phenotype (Hume et al., 2020).

AIRE plays a key role in central tolerance by regulating the expression of peripheral tissue antigens in epithelial cells of the thymus and by eliminating autoreactive T cells. Patients with the autoimmune polyendocrinopathy-candidiasis-ectodermal-dystrophy syndrome have genetic defects in AIRE. *Aire* KO rats show signs of generalized autoimmunity and clinical signs of disease that are much more pronounced than in *Aire* KO mice and closer to manifestations in humans (Ossart et al., 2018).

## Conclusion and Perspectives

CRISPR-Cas system is now the tool of choice for genome editing, particularly for the rat for which ES cells are limited compared to the mouse. In the last decade, efforts have been made to improve this tool and its delivery but two main hurdles persist. Some loci are still difficult or impossible to edit, and the efficiency of large or complex KI is still too low. Although many advances have been developed in the application of the CRISPR-Cas system to human cells and sometimes in mice, many remain to be applied in rat model generation.

Rats often proved to be better mimics of human situation than mice. It is particularly evident in CVD, neurobiology, ophthalmology, muscular diseases, and immunology, but few of the large number of genetic diseases in these or other organ systems have been modeled in rats. It is difficult to predict when the rat will be better than the mouse, nevertheless, it seems reasonable to try to generate new genetically modified rats in these areas. Moreover, to the best of our knowledge and among the models that can be compared, there are no mouse genetic or immune models that better reproduce human disease than rat. Future work using the CRISPR-Cas system will likely generate new rat models of genetic diseases and to study genes functions. Extensive work in QTLs associated with major polygenic diseases has been performed in rats (Gauguier, 2016; Shimoyama et al., 2017). Within these QTLs, the genes that could be responsible for a given disease will likely be targets of choice in future studies.

Other genes that would be logical to target in rats are those that are absent in mice and present in humans, given that 78 out of the 2544 Mb of the rat genome is common between humans and rats but not humans and mice (Gibbs et al., 2004). Examples within the immune system include *Tlr10* and *Cd89*.

A limitation of rats versus mice that cannot be resolved is also one of its advantages: its bigger size, which brings higher breeding costs.

The rat will continue to be a critical experimental model based on its bigger size and its inherent physiological characteristics, as well as a large and growing body of physiology and genomic data. Tools for modifying the rat genome as well as analyzing the genome are key to the development of new models for understanding biology and diseases.

## Author Contributions

All authors performed the bibliographic research and participated in writing the manuscript. IA planned the review and secured the funding.

## Conflict of Interest

YC and VC are genOway employees. The remaining authors declare that the research was conducted in the absence of any commercial or financial relationships that could be construed as a potential conflict of interest.
